# The SnRK2.10 kinase mitigates the adverse effects of salinity by protecting photosynthetic machinery

**DOI:** 10.1093/plphys/kiab438

**Published:** 2021-09-23

**Authors:** Radosław Mazur, Justyna Maszkowska, Anna Anielska-Mazur, Maciej Garstka, Lidia Polkowska-Kowalczyk, Anna Czajkowska, Agnieszka Zmienko, Grazyna Dobrowolska, Anna Kulik

**Affiliations:** 1 Department of Metabolic Regulation, Institute of Biochemistry, Faculty of Biology, University of Warsaw, Miecznikowa 1, 02-096 Warsaw, Poland; 2 Institute of Biochemistry and Biophysics, Polish Academy of Sciences, Pawińskiego 5a, 02-106 Warsaw, Poland; 3 Warsaw University of Life Sciences – SGGW, Nowoursynowska 166, 02-787 Warsaw, Poland; 4 Institute of Bioorganic Chemistry, Polish Academy of Sciences, Noskowskiego 12/14, 61-704 Poznań, Poland

## Abstract

SNF1-Related protein kinases Type 2 (SnRK2) are plant-specific enzymes widely distributed across the plant kingdom. They are key players controlling abscisic acid (ABA)-dependent and ABA-independent signaling pathways in the plant response to osmotic stress. Here we established that SnRK2.4 and SnRK2.10, ABA-nonactivated kinases, are activated in *Arabidopsis thaliana* rosettes during the early response to salt stress and contribute to leaf growth retardation under prolonged salinity but act by maintaining different salt-triggered mechanisms. Under salinity, *snrk2.10* insertion mutants were impaired in the reconstruction and rearrangement of damaged core and antenna protein complexes in photosystem II (PSII), which led to stronger non-photochemical quenching, lower maximal quantum yield of PSII, and lower adaptation of the photosynthetic apparatus to high light intensity. The observed effects were likely caused by disturbed accumulation and phosphorylation status of the main PSII core and antenna proteins. Finally, we found a higher accumulation of reactive oxygen species (ROS) in the *snrk2.10* mutant leaves under a few-day-long exposure to salinity which also could contribute to the stronger damage of the photosynthetic apparatus and cause other deleterious effects affecting plant growth. We found that the *snrk2.4* mutant plants did not display substantial changes in photosynthesis. Overall, our results indicate that SnRK2.10 is activated in leaves shortly after plant exposure to salinity and contributes to salt stress tolerance by maintaining efficient photosynthesis and preventing oxidative damage.

## Introduction

Salinity and water deficiency are common environmental factors limiting plant productivity and affecting agricultural yield (Acosta-Motos et al., 2017). A proper recognition of stress conditions, triggering adequate signaling pathways, and ensuring adjustment of metabolic programs are critical for optimization of growth, reproduction, and survival under severe conditions. Stress signals are recognized and transmitted to different cellular compartments by specialized signaling pathways in which protein kinases and phosphatases are key components. Among the protein kinases involved in stress signal transduction in plants, members of the SNF1-related kinases (SnRKs) family have been under intensive study in recent years. Plant SnRKs are classified into the SNF1/AMPK family, which also comprises the sucrose non-fermenting-1 (SNF1) kinase from the yeast and mammalian AMP-activated protein kinase (AMPK). SnRK2s are plant-specific protein kinases with a molecular mass of about 40 kDa identified in *Arabidopsis thaliana* (Boudsocq et al., 2004) and in many other plant species like rice, sorghum, maize, tobacco, tomato, wheat, soybean, fava bean, and algae (for review see Kulik et al., 2011; Shinozawa et al., 2019). The SnRK2 family kinases have been divided into three groups: group 1—kinases not activated by ABA, group 2—weakly activated, and group 3—strongly activated by ABA (for review see Boudsocq and Laurière, 2005; Kulik et al., 2011). In *A. thaliana*, the SnRK2 kinase family consists of 10 members. Among them, SnRK2.1, 2.4, 2.5, 2.9, and 2.10 belong to group 1, SnRK2.7 and 2.8 to group 2, and SnRK2.2, 2.3, and 2.6 to group 3. All of them, except SnRK2.9, are rapidly activated in response to osmotic stress (for review see Kulik et al., 2011). According to a recent study, group 3 SnRK2s are the ancient form of land-plant SnRK2s, whereas group 1 is the most recent one which has evolved in vascular plants (Shinozawa et al., 2019).

The best-characterized SnRK2s in Arabidopsis are the ABA-dependent ones. A triple knockout mutant *snrk2.2/2.3/2.6* is completely insensitive to ABA and exhibits greatly reduced tolerance to drought as a consequence of impaired stomatal closure and downregulation of ABA- and water stress-induced genes (Fujii and Zhu, 2009; Fujita et al., 2009; Yoshida et al., 2015; for review see Fujita et al., 2013). The function of these three kinases is partially redundant, however, SnRK2.2 and SnRK2.3 are mainly involved in the regulation of seed dormancy and germination (Fujii et al., 2007), while SnRK2.6 is involved in ABA-dependent stomatal movements (Mustilli et al., 2002). SnRK2.7 and SnRK2.8 kinases, belonging to group 2, play significant roles in improving the drought resistance of *A. thaliana* by controlling primary and lateral root growth and biomass accumulation under nutrient deprivation (Umezawa et al., 2004; Shin et al., 2007).

The understanding of the physiological role of group 1 SnRK2s is the most limited. Besides hyperosmotic stress, they are activated in response to cadmium and copper ions, the fungal elicitor cryptogein, nitric oxide, and reactive oxygen species in Arabidopsis and tobacco cells (Lamotte et al., 2006; Wawer et al., 2010; Kulik et al., 2012, 2014). Recently, it has been shown that ABA-non activated SnRK2s phosphorylate varicose (VCS), an mRNA decapping activator, and thus regulate mRNA decay under osmotic and salt stress conditions (Soma et al., 2017; Kawa et al., 2020). Two dehydrins, Early Responsive to Dehydration 10 and 14 (ERD10 and ERD14), were identified as direct substrates of SnRK2.10 (Maszkowska et al., 2019). SnRK2.4 regulates primary root growth, whereas SnRK2.10 controls lateral root density under salt stress (McLoughlin et al., 2012). Group 1 SnRK2s function in root development under salt stress by affecting, among others, the transcript levels of aquaporins *PIP2;*3 and *PIP2;5*, and *CYP79B2*, a gene coding enzyme involved in auxin biosynthesis (Kawa et al., 2020). Redundancy within the group 1 SnRK2s has been suggested regarding the regulation of root architecture in non-stress conditions (Kawa et al., 2020). It has also been established that SnRK2.10 and/or SnRK2.4 regulate, by an unknown mechanism, reactive oxygen species (ROS) homeostasis in plants exposed to heavy metal stress or salinity (Kulik et al., 2012; Szymańska et al., 2019).

The plant response to stressful conditions requires a high consumption of energy. Moreover, stress often leads to energy deficit, mainly through the limitation of photosynthesis. Photosynthesis, and subsequently cell growth, are among the primary processes affected by drought and by salinity (Chaves et al., 2009). Salinity may influence photosynthesis in several ways.

First, it causes a deficit of water which is required for maintaining of proper biochemical environment for all physiological and biochemical processes occurring in the plant cell, including its role as the electron donor in the light phase of photosynthesis. Second, high concentrations of ions, such as Na^+^ and Cl^–^, which accumulate in chloroplasts under salinity stress, damage thylakoid membranes and thus disrupt photosynthetic reactions (Chaves et al., 2009). Next, closing of stomata is one of the first defense mechanisms against drought or salinity which results in limitation in CO_2_ and oxygen availability. Salt and drought stress diminish the activity of several dark phase photosynthesis enzymes (e.g. Rubisco, sucrose-phosphate synthase, fructose-1,6-bisphosphatase, and phosphoenolpyruvate carboxylase) in various plant species (for review see Chaves et al., 2009). Moreover, salinity strongly reduces the level of chlorophylls and carotenoids. Further, under unfavorable environmental conditions photosystems may undergo over-excitation leading to overproduction of ROS in chloroplasts. ROS, such as singlet oxygen (^1^O_2_), superoxide anion (O_2_^•−^), and H_2_O_2_, are considered major factors responsible for the injury of the photosynthetic apparatus and diminishing of photosynthesis efficiency (for review see Asada, 2006; Foyer and Shigeoka, 2011). As a consequence of salinity-caused injury of the photosynthetic apparatus the chlorophyll fluorescence alters. Changes in the chlorophyll fluorescence induction parameters like the *F*_v_/*F*_m_ ratio, NPQ, Y(I), Y(II), and others are widely observed when photosynthetic disorders occur (Baker and Rosenqvist, 2004). Reduction of the photosynthesis rate under environmental stress diminishes ATP production and soluble sugar content which are the primary energy sources for metabolism and stress defense.

The photosynthesis efficiency is strictly correlated with biomass production and seed yield under salt stress. This makes studies on the regulation of photosynthesis under water deficiency and salinity extremely important from the agricultural, economic, and ecological points of view. The role of ABA-nonresponsive SnRK2s in the response of leaves to salinity has been studied only sketchily. Here we show that SnRK2.4 and SnRK2.10 are activated in Arabidopsis rosettes shortly after exposition of plants to salinity and contribute to leaf growth retardation under prolonged stress. However, the mode of action of these closely related kinases seems to be different. SnRK2.10 is required to maintain high photosynthesis efficiency and contributes to ROS homeostasis during long-term salt stress, while SnRK2.4 seems to control other, still unknown salt-dependent events.

## Results

### SnRK2.4 and SnRK2.10 are activated in leaves in response to salt stress

**Figure 1:**
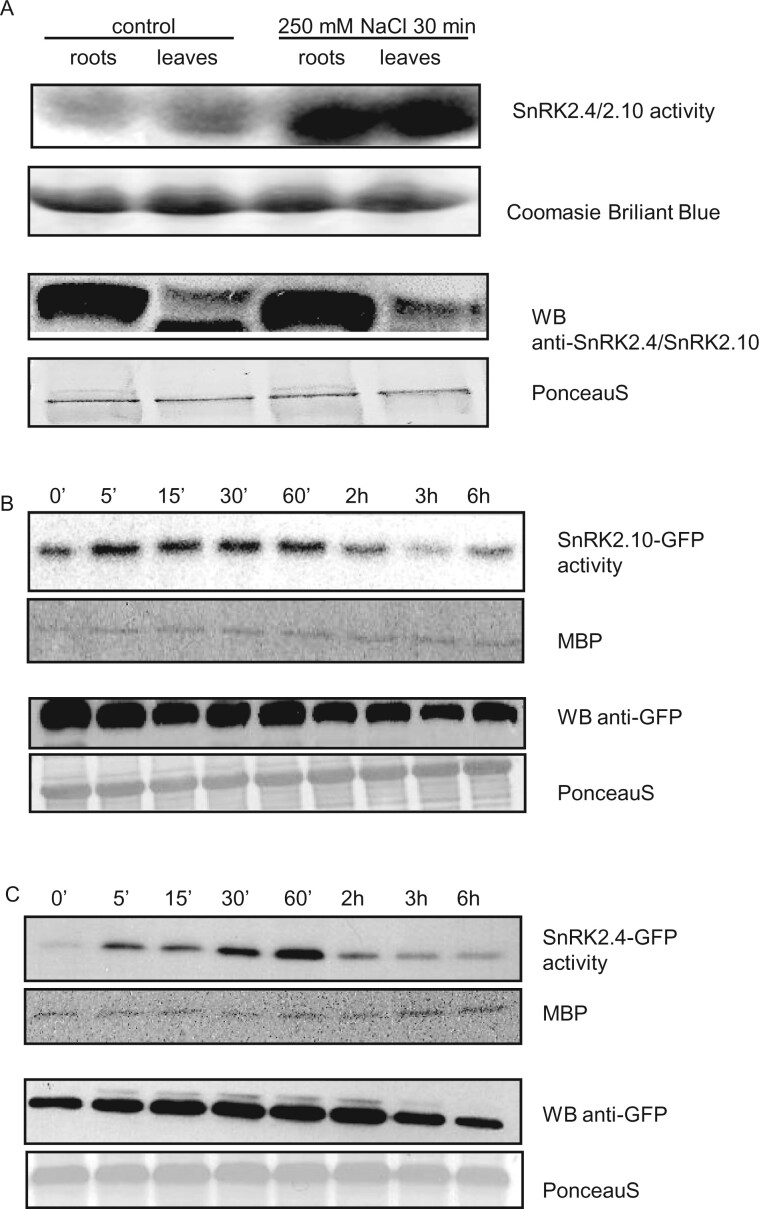
Activation of SnRK2.4 and SnRK2.10 kinases in Arabidopsis leaves upon salt stress. *A. thaliana* plants of wild-type Col-0 and lines expressing SnRK2.4-GFP or SnRK2.10-GFP were grown in hydroponic culture for 5 weeks and then transferred to medium supplemented with 250 mM NaCl for up to 6 h. Rosettes were collected, homogenized, and protein kinases immunoprecipitated with antibodies recognizing SnRK2.4/SnRK2.10 kinases (A) or GFP (B and C). Kinase activity of immunoprecipitated proteins was determined by in-gel kinase assay (A) or in solution assay (B and C) with γ-[^32^P] ATP and MBP (myelin basic protein) as substrates. The amount of SnRK2.4 and SnRK2.10 was determined by western blotting with anti-SnRK2.4/SnRK2.10 antibodies (A) and SnRK2.4-GFP and SnRK2.10-GFP were determined by western blotting with anti-GFP antibodies (B and C). The figure presents a representative result of three independent repeats of the experiment.

It has been shown previously that SnRK2 protein kinases belonging to group 1 are rapidly activated in plant protoplasts, roots, or whole seedlings exposed to NaCl (Boudsocq et al., 2004; McLoughlin et al., 2012; Soma et al., 2017). To check whether these kinases are also activated in leaves when only the roots are exposed to salinity, Arabidopsis plants were grown in a hydroponic system and treated with 250 mM NaCl. Protein kinase activity was monitored separately in roots and rosettes by an in-gel activity assay after immunoprecipitation of SnRK2.4/2.10 by specific antibodies recognizing an N-terminal fragment of both kinases as described previously (Kulik et al., 2012). As presented in Figure 1, A, the SnRK2.4 and SnRK2.10 kinase(s) were active in roots and rosettes after the plant treatment with salt for 30 min. In control samples (not treated with NaCl), only slight activity was observed. The level of both proteins stayed unchanged upon treatment but was relatively lower in Arabidopsis rosettes comparing with roots (Figure 1, A). Since SnRK2.4 and SnRK2.10 have been shown to play lightly different roles in the response to water regime and salinity, despite their high amino acid sequence similarity (McLoughlin et al., 2012; Maszkowska et al., 2019; Szymańska et al., 2019), we decided to monitor the activity of each enzyme separately. For this purpose, we used plants expressing SnRK2.4 or SnRK2.10 in fusion with GFP. Hydroponically grown plants were treated with 250 mM NaCl for various periods of time (up to 6 h) and the kinase activity was monitored after immunoprecipitation using anti-GFP antibodies. Both SnRK2.4 and SnRK2.10 were transiently activated in Arabidopsis leaves in response to salinity (Figure 1, B and C). The activation occurred after 5 min of salt application and lasted for up to 1 h. In parallel, the protein levels of SnRK2.4-GFP and SnRK2.10-GFP were monitored by Western blotting with specific anti-GFP antibodies and show only slight fluctuation between samples.

### Both SnRK2.4 and SnRK2.10 contribute to plant growth under salinity stress

**Figure 2:**
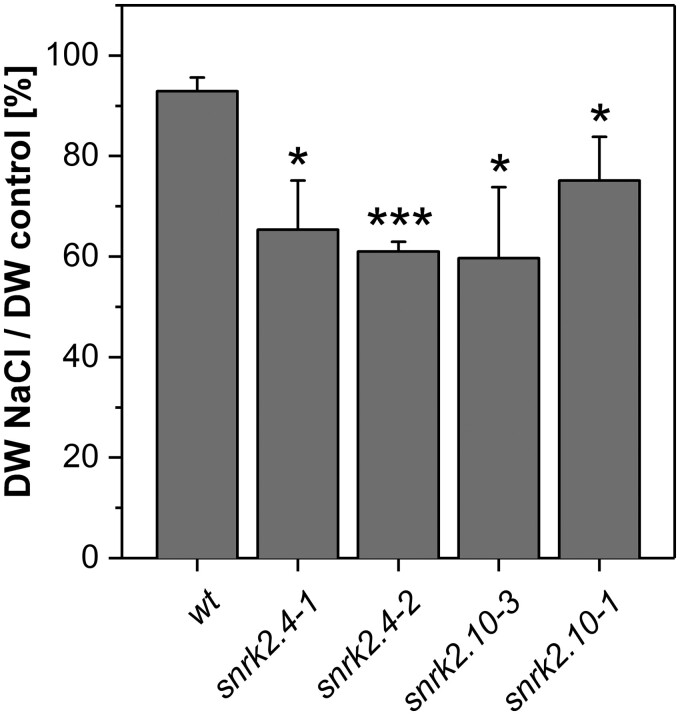
Effect of long-term salt stress on rosette dry mass in *snrk2.4 and snrk2.10* mutant lines. *Arabidopsis thaliana* plants of wt Col-0 and two independent *snrk2.4 and snrk2.10* insertion mutant lines each were grown in hydroponic culture for 5 weeks and then moved to medium with or without (control) 150 mM NaCl for next 6 d. Dry weight (DW) of salt-stressed rosettes was compared with that of control plants. Mean ± SD values from three independent experiments are shown, with 10 plants per line per condition in each experiment. Statistical significance of the difference between a mutant line and wt was determined using Student’s *t*-test; *P<0.05; **P<0.01; ***P<0.001.

Salt stress strongly impairs plant growth. So far it has been shown that under salinity SnRK2.10 regulates the formation of lateral roots while SnRK2.4 controls primary root elongation (McLoughlin et al., 2012) and thus both kinases are important for the underground organ growth rate. To gain more insight into the role of the two SnRK2s in the response of Arabidopsis leaves to salinity, we analyzed the NaCl-response phenotypes of two independent lines of *snrk2.4* (*snrk2.4-1 and snrk2.4-2*) and *snrk2.10* (*snrk2.10-1 and snrk2.10-3*) knockout mutants. We determined the dry weight of rosettes from plants grown for 5 weeks in a control hydroponic medium followed by 6 d in the medium supplemented or not (control) with 150 mM NaCl. In wild-type (wt) plants treated with salt, the dry weight of rosettes was reduced to about 90% of the value for control plants (Figure 2). In the *snrk2.4-1 and snrk2.4-2* mutants, the salinity reduced dry weight to about 65% and 61%, respectively, and in *snrk2.10-3 and snrk2.10-1* to, respectively, about 59% and 75% of the respective control values, indicating that the growth of the mutants was more strongly affected by salinity than the growth of wt plants.

Measurement of stomatal conductance revealed that after already 1 h of treatment with NaCl, water permeability in all leaves dropped to half of its baseline value and longer treatment caused an additional reduction to about 20% (Supplemental Figure S1A). Our results showed that there was no significant difference between plant lines tested in stomatal conductance and stomata index (Supplemental Figure S1, A–C). In parallel, *SnRK2.4 and SnRK2.10* expression was monitored in wt plants (Supplemental Figure S2) during the exposure to salinity. The expression of *SnRK2.4* was stable throughout the salt treatment while *SnRK2.10* expression was slightly reduced on the sixth day.

### SnRK2.10 signaling pathway maintains photosynthesis efficiency under salinity stress

**Figure 3:**
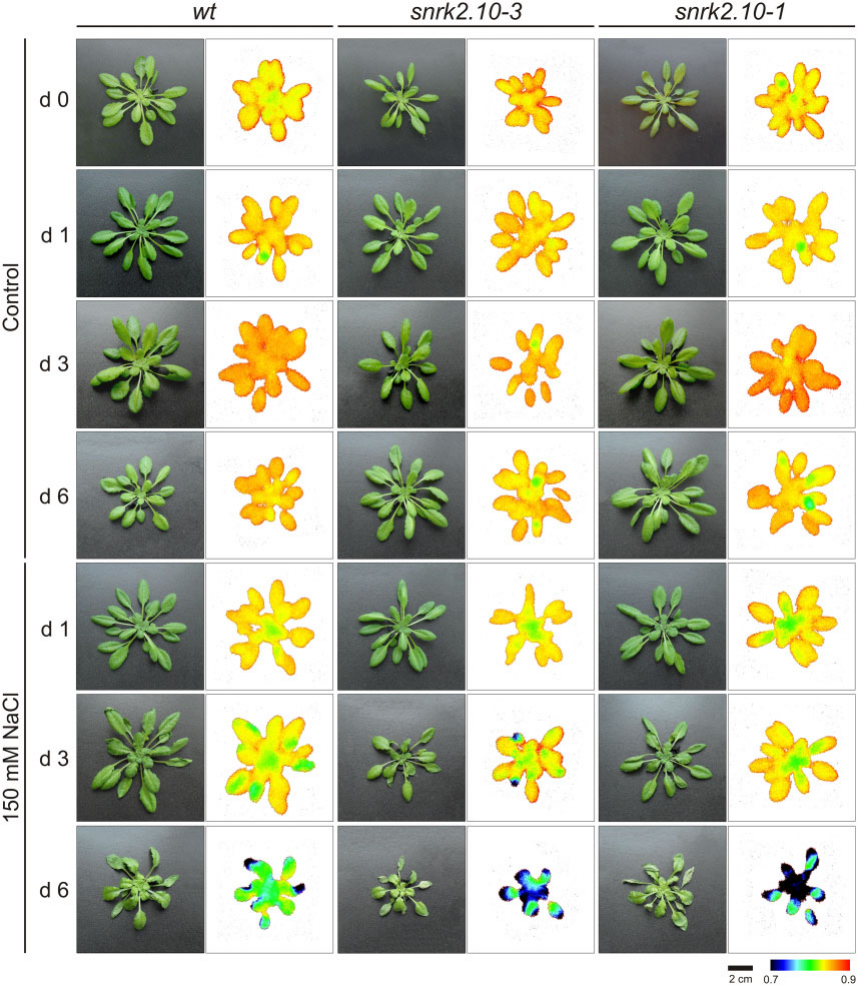
Effect of salt stress on chlorophyll *a* fluorescence distribution of wt and *snrk2.10* mutant plants. Pictures present leaf morphology (left-hand columns) and *F*_V_/*F*_M_ ratio (right-hand columns) for plants grown in standard medium (control) and plants treated with 150 mM NaCl for up to 6 d. The images are representative of at least four rosettes from each treatment.

Using transgenic plants expressing either pSnRK2.4::SnRK2.4-YFP or pSnRK2.10::SnRK2.10-YFP we found that both, SnRK2.4 and SnRK2.10 kinases were present in the cytoplasm of cells in different leaf tissues, including the palisade mesophyll (Supplemental Figure S3), the main site for photosynthesis. To investigate the impact of SnRK2.10 and SnRK2.4 on the response of the photosynthetic apparatus to salt stress, we compared several chlorophyll fluorescence parameters in wt, *snrk2.10*, and *snrk2.4* mutant plants grown under control and salt stress conditions. The maximal quantum yield of photosystem II (PSII; *F*_V_/*F*_M_) in control conditions was not affected by the *snrk2.10* knockout and was similar to that of the wt, above 0.8, a value typical for plants grown in optimal conditions (Murchie and Lawson, 2013); it did not change during the 6 d of the experiment. In contrast, in plants exposed to 150 mM NaCl *F*_V_/*F*_M_ was decreasing in time in all plant lines, and in the *snrk2.10-1 and snrk2.10-3* lines, this decrease was much stronger than in the wt plants (Figure 3). A lower concentration of NaCl (100 mM) did not induce substantial *F*_V_/*F*_M_ changes (Supplemental Figure S4).

**Figure 4:**
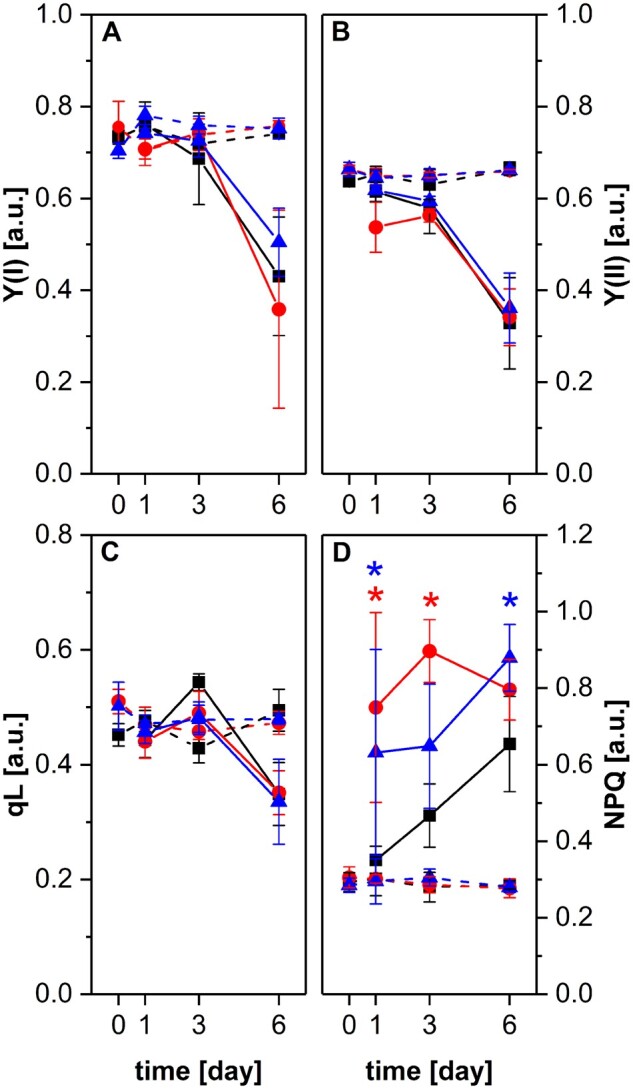
Effect of salt stress on efficiency of photosynthetic light reactions of wt and *snrk2.10* mutant plants under steady light intensity. Photosynthetic parameters: Y(I) (A), Y(II) (B), qL (C), and NPQ (D) were determined under steady light conditions for wt (black squares), *snrk2.10-3* (red circles), and *snrk2.10-1* (blue triangles) plants grown under control conditions (dashed lines) and treated with 150 mM NaCl (solid lines). Data are means ± SD from three independent experiments. Statistical significance of difference was determined using ANOVA followed by post hoc Tukey’s test (*P*<0.05). Asterisks mark the statistical significance of difference between a mutant line and wt plants.

The decrease of *F*_V_/*F*_M_ indicated that the photochemical activity was impaired in salt-treated plants, therefore we analyzed other chlorophyll fluorescence parameters to establish the nature of that impairment. In actinic light of constant intensity, the photochemical yields of PSI (Y(I)) and PSII (Y(II)) decreased with the duration of exposure to 150 mM NaCl (starting from Day 1) without differences between the genotypes (Figure 4 and Supplemental Figure S5, A). Also, photochemical quenching (qL) changed in a similar pattern in all three lines studied, showing a constant drop up to the sixth day of salt treatment (Figure 4C and Supplemental Figure S5, B). In contrast, non-photochemical quenching (NPQ) showed markedly different behavior between wt and the *snrk2.10* mutants. In the wt plants, the salt treatment produced initially (Day 1) a very modest increase of NPQ which progressed steadily until Day 6. In the two *snrk2.10* lines, the initial increase of NPQ was much pronounced (ca. 2-fold compared to control growth conditions) and then showed only a moderate increase or, in one of the lines, a slight drop between Days 3 and 6 (Figure 4, D). Still, in both mutant lines, the NPQ value was significantly higher than in the wt. At 100 mM NaCl, the analyzed parameters did not change significantly except for NPQ, which increased after 6 d of treatment in both *snrk2.10* lines but not in wt plants; after the next 3 d, the NPQ values for the *snrk2.10* lines decreased to the level observed in wt plants (Supplemental Figure S6). Additionally, we found that impairment in photosynthesis parameters was comparable in the wt and *snrk2.10* plants treated with 150 g/L PEG 8000 what suggested that maintenance of photosynthesis efficiency by SnRK2.10-dependent pathways is a salt specific response (Supplemental Figure S5).

**Figure 5:**
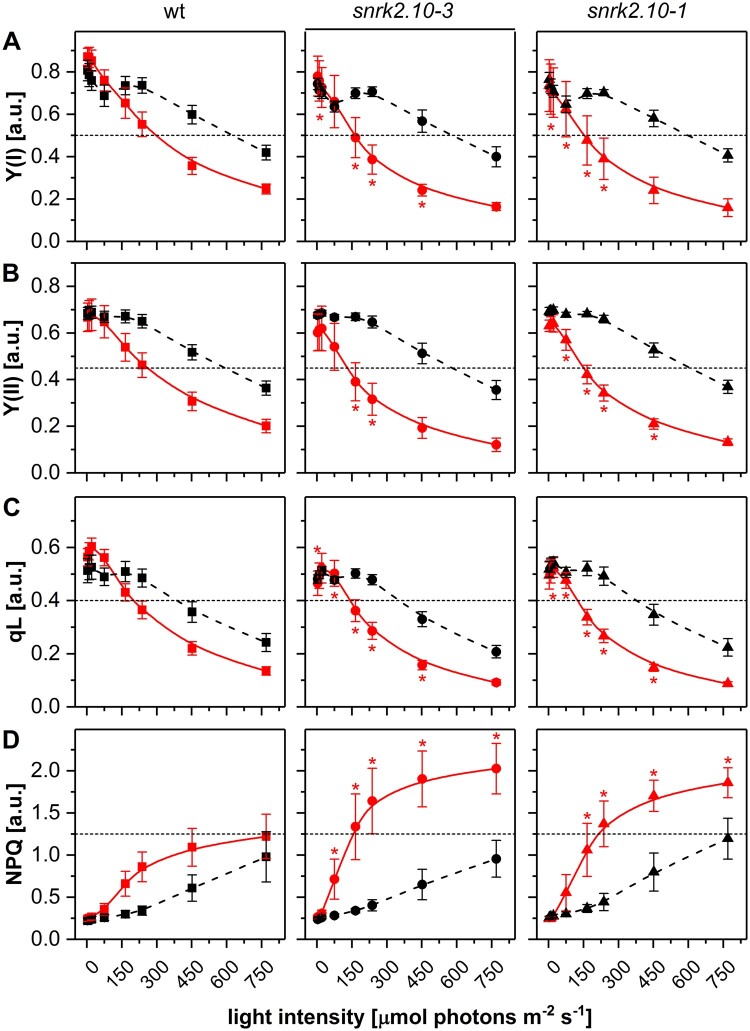
Effect of salt stress on efficiency of photosynthetic light reactions of wt and *snrk2.10* mutant plants under increasing light intensity. Photosynthetic parameters: Y(I) (A), Y(II) (B), qL (C), and NPQ (D) measured for plants grown in control conditions (black dashed lines) and after treatment with 150 mM NaCl for 3 d (red solid lines). Data are means ± SD from three independent experiments. Statistical significance of difference was determined using ANOVA followed by post hoc Tukey’s test (*P*<0.05). Asterisks mark the statistical significance of difference between a mutant line and wt plants.

**Figure 6:**
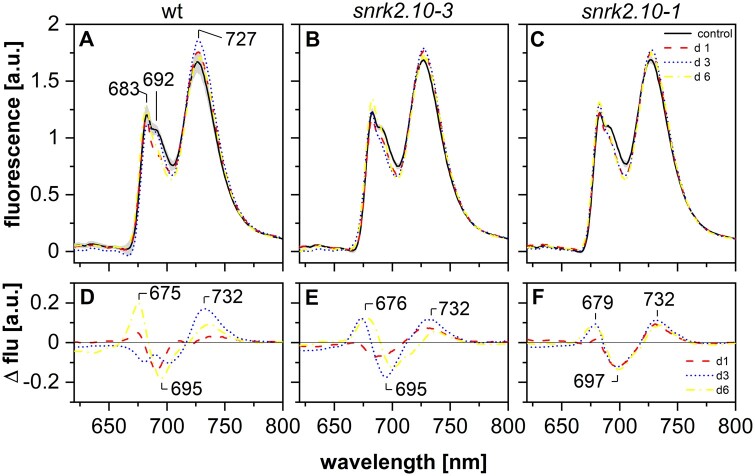
Salinity-dependent changes in chlorophyll *a* fluorescence emission spectra in isolated thylakoids of wt and *snrk2.10* mutant plants. Fluorescence of chlorophyll *a* was excited at 440 nm for thylakoids isolated from control plants (black solid line) and plants subjected to salt treatment (150 mM NaCl) for 1 d (red dashed line), 3 d (blue dotted line), or 6 d (yellow dash-dotted line). Difference spectra (lower panels) were calculated by subtracting the control spectrum from respective spectra for salt-treated plants; color scheme as above. The presented spectra are representative of three independent experiments. A and D data set for wt plants, B and E – data set for *snrk2.10-3* mutant, C and F – data set for *snrk2.10-1* mutant.

We then analyzed the same photosynthetic parameters using a different illumination mode with the light intensity increasing exponentially during the assay. Such an approach allows determining the plant response to the rapid changes of light conditions. To simplify the analysis, only plants subjected to salt stress for 3 d were compared with those grown in control conditions. With increasing actinic light intensities the values of Y(I), Y(II), and qL decreased (Figure 5, A–C). For plants grown under control conditions, the route of that decrease was fairly moderate and virtually identical for all three lines. For salt-treated plants, the rate of decrease of all three parameters was higher than for control ones and, notably, markedly higher in the two *snrk2.10* mutants than in the wt. The difference between the wt and *snrk2.10* lines was even more pronounced for NPQ (Figure 5D). In wt plants, the salt treatment only slightly affected the rate and extent of NPQ increase with increasing actinic light intensities, whereas in the two *snrk2.10* lines the difference between salt-treated and control plants was much stronger. As was the case for the steady light conditions, the two *snrk2.10* lines showed slightly different behavior. These results indicate that in plants exposed to salinity stress photosystems I and II are under a high excitonic pressure and are unable to efficiently utilize light energy, and this effect is markedly stronger in the *snrk2.10* mutant compared to wt plants.

We also tested the influence of 150 mM NaCl treatment on the photosynthetic performance of *snrk2.4* mutants (Supplemental Figure S7). The decrease of *F*_V_/*F*_M_, Y(I), Y(II), and qL measured in steady light conditions was lower in the two *snrk2.4* lines compared to wt plants (Supplemental Figure S7, A–C and E). Moreover, there were no differences in the photosynthetic parameters measured using increasing actinic light intensities between *snrk2.4* and for wt plants subjected to salt stress for 3 d (Supplemental Figure S7, F–I). These results show a fundamentally different response of the *snrk2.10 and snrk2.4* mutants to salinity stress.

**Figure 7:**
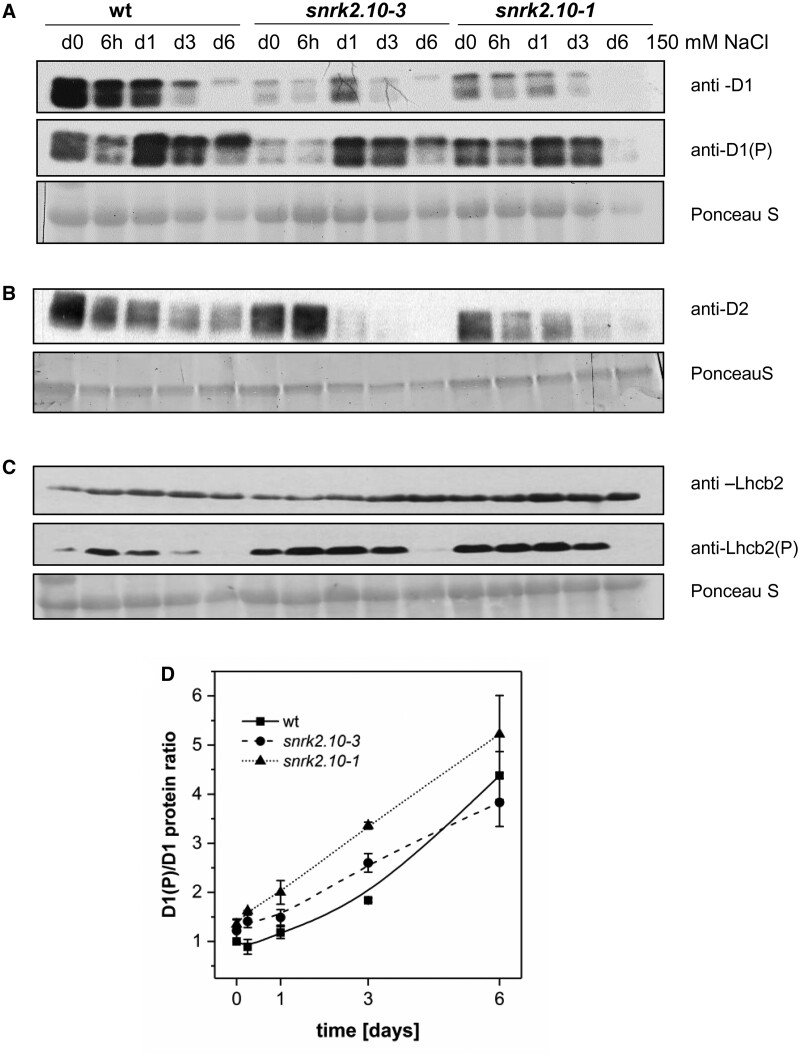
SnRK2.10 affects levels of D1, D2, and Lhcb2 proteins and their phosphorylation status in plants subjected to salt stress. Proteins were extracted from rosettes of wild type and *snrk2.10* mutant plants treated with 150 mM NaCl for up to 6 d. Monitoring of proteins levels was conducted by Western blotting using specific antibodies recognizing D1 and D1(P) (phosphorylated D1 form) proteins (A); D2 protein (B); Lhcb2 and Lhcb2(P) (phosphorylated form of Lhcb2) proteins (C). Figure presents results one from three replicates of experiment showing similar results. D, Relative extent of protein phosphorylation was determined by dividing signal intensity for the phosphorylated form of D1 by that for total protein. D1(P)/D1 signal intensity ratio in wt d0 was considered as 1. Mean values ± SD for three independent experiments showing similar results are shown.

As the above results indicated that SnRK2.10-dependent signaling rather than the SnRK2.4-dependent one is involved in the regulation of photosynthesis efficiency under long-term salinity, we focused further studies on this kinase.

### SnRK2.10 affects organization of chlorophyll–protein complexes but not composition of photosynthetic pigments in plants subjected to salinity stress

The organization of chlorophyll–protein complexes in photosynthetic apparatus is changing under environmental stress because of its injury and/or as an acclimation for functioning in new unfavorable conditions. These changes are associated with modifications of chlorophyll fluorescence emission spectra at specific wavelengths. To compare the contribution of specific chlorophyll–protein (CP) complexes to the overall fluorescence pattern in thylakoids isolated from wt and *snrk2.10* plants exposed to salt stress, the steady-state chlorophyll fluorescence emission spectra at 77 K were determined (Figure 6). The spectra for wt, *snrk2.10-3 and snrk2.10-1* plants grown in control conditions (Figure 6, A–C, black solid line) were virtually identical, with typical features for the chlorophyll fluorescence emission spectrum of higher plants composed of two bands centered at 683 nm and 727 nm and related to the fluorescence from PSII-LHCII and PSI-LHCI, respectively. The PSII-LHCII band showed a shoulder around 692 nm related to the PSII core complex (Andreeva et al., 2003; Rumak et al., 2012).

The exposure of plants to 150 mM NaCl, changed the emission spectrum of thylakoids, especially in the PSII-related region (Figure 6, A–C, dashed, dotted, and dash-dotted lines). To quantify the changes, differential spectra were calculated for plants treated with150 mM NaCl for 1, 3, and 6 d and compared to control plants (Figure 6, D–F). In wt thylakoids from plants exposed to salt for 1 d, there was a negative band centered at around 690 nm related to PSII reaction centers and inner PSII antennae (Figure 6, D). After 3 d of exposure, a positive band at 732 nm and two negative bands at around 680 and 700 nm were visible (Figure 6, D). After 6 d differential, the spectra showed positive bands centered at 675 nm, corresponding to trimeric LHCII disconnected from PSII (Kovacs et al., 2006), at 732 nm, corresponding to PSI-LHCI supercomplexes, and a negative band centered at 695 nm, corresponding to PSII reaction centers (Figure 6, D). In the *snrk2.10* mutants (Figure 6, E and F), the shape of the difference spectra was similar to those for wt plants, showing positive bands at around 675 and 732 nm and a negative one at around 695 nm after 3 and 6 d of treatment but, the amplitude of the spectra was slightly lower compared to wt plants.

These results suggest that the treatment of plants with 150 mM NaCl decreased the amount of PSII core complexes and increased the amount of trimeric LHCII complexes disconnected from PSII-LHCII. Also, the content of PSI-LHCI in thylakoids increased. These changes were less pronounced in *snrk2.10* mutants than in wt plants.

An analysis of the chlorophyll and carotenoid content in chloroplasts from wt and *snrk2.10* plants showed that the abundance and proportions of those pigments change in a complex manner during salinity stress and the changes are very similar in all three lines studied (Supplemental Figure S8 and Supplemental Results). Thus, one may conclude that SnRK2.10 is not involved in the regulation of chlorophylls and carotenoids composition upon salt stress.

**Figure 8:**
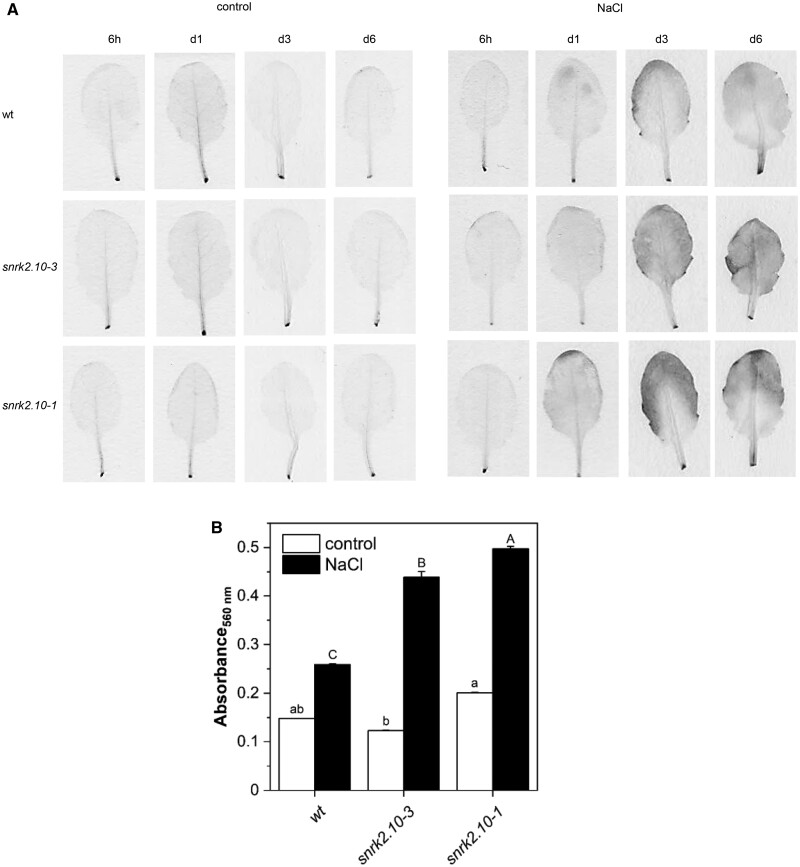
SnRK2.10 affects accumulation of H_2_O_2_ in *A. thaliana* leaves under long-term salinity. Six-week-old plants of wild type and *snrk2.10* mutant were treated with 150 mM NaCl or kept in control media for up to 6 d. Leaves of a similar age were collected at indicated times and stained for H_2_O_2_ with DAB (A). After removal of photosynthetic and nonphotosynthetic pigments, leaves were photographed to show DAB staining. B, Leaves treated or not with 150 mM NaCl for 6 d were powdered and H_2_O_2_ detected by reaction with Amplex Red dye. Figure presents results one from three replicates of experiment showing similar results with 8–10 leaves used per line and treatment in every biological repetition. Bars represent mean ± SD. For statistical analysis, ANOVA followed by Tukey test were applied (*P*<0.05). Statistical analysis was done separately for control (lowercases littering) and salt-treated (lowercases littering) samples.

### SnRK2.10 contributes to the regulation of the abundance and phosphorylation status of selected PSII and LHCII proteins

The PSII core is considered the photosynthetic machinery component most susceptible to diverse abiotic stresses, easily undergoing photoinhibition (Gururani et al., 2015; Liu et al., 2019). During the response to various unfavorable environmental conditions individual PSII center proteins are cyclically damaged and repaired in a defined sequence of events comprising their phosphorylation and dephosphorylation, disassembly of the PSII complex, proteolysis and *de novo* synthesis of D1 protein, and finally reconstitution of the complex (Gururani et al., 2015; Lu, 2016). Also, LHCII proteins undergo controlled phosphorylation and dephosphorylation which allows the state transition in LHCII, rearrangement of protein complexes, and adjustment of the photosynthetic machinery to stress conditions. Because the chlorophyll *a* fluorescence data suggested a different response of PSII core complex components to salt treatment in *snrk2.10* mutants compared to wt plants, we monitored the levels of two PSII core proteins, D1 and D2, in those lines. Salinity gradually diminished the content of D1 and D2 proteins in both the wt and the *snrk2.10* lines, although *snrk2.10-3* showed a transient accumulation of D1 at Day 1 and of D2 at 6 h of treatment (Figure 7, A and B). Notably, the level of D1 was markedly and of D2, slightly, lower in the *snrk2.10-1* line compared to the wt at the beginning of salt treatment. Unlike the global level of D1, the content of its phosphorylated form actually increased, upon salinity so that the fraction of D1 that was phosphorylated increased fairly uniformly throughout the 6 d of salt treatment; no significant differences in the course of this increase were observed between the wt and the *snrk2.10* lines, although between 6 h and 3 d, the relative level of D1 phosphorylation was slightly higher in the mutants (Figure 7D).

In contrast, the level of Lhcb2 (light-harvesting chlorophyll *b* binding 2) protein was only slightly affected by salinity: in the wt, it increased a little upon salt application and then did not change, while in the *snrk2.10* mutants an upwards tendency was seen throughout the treatment (Figure 7C). The content of phosphorylated Lhcb2 increased strongly upon salt application in the wt and then went down, while in the *snrk2.10* lines a minimal increase was observed only upon salt application and then a total loss between Days 3 and 6. Moreover, since in control conditions, the extent of Lhcb2 phosphorylation was markedly higher in the mutants than in wt, its overall content remained substantially higher compared to wt between Days 0 and 3.

Higher phosphorylation of Lhcb2 in *snrk2.10* plants may disturb the state transition of LHCII protein complexes under salinity. Observed disturbances in D1 and Lhcb2 proteins levels and their phosphorylation status were specific to SnRK2.10-dependent pathways and did not rely on D1/Lhcb2 cross-affecting (please see Supplemental Figure S9 and Supplemental Results).

**Figure 9:**
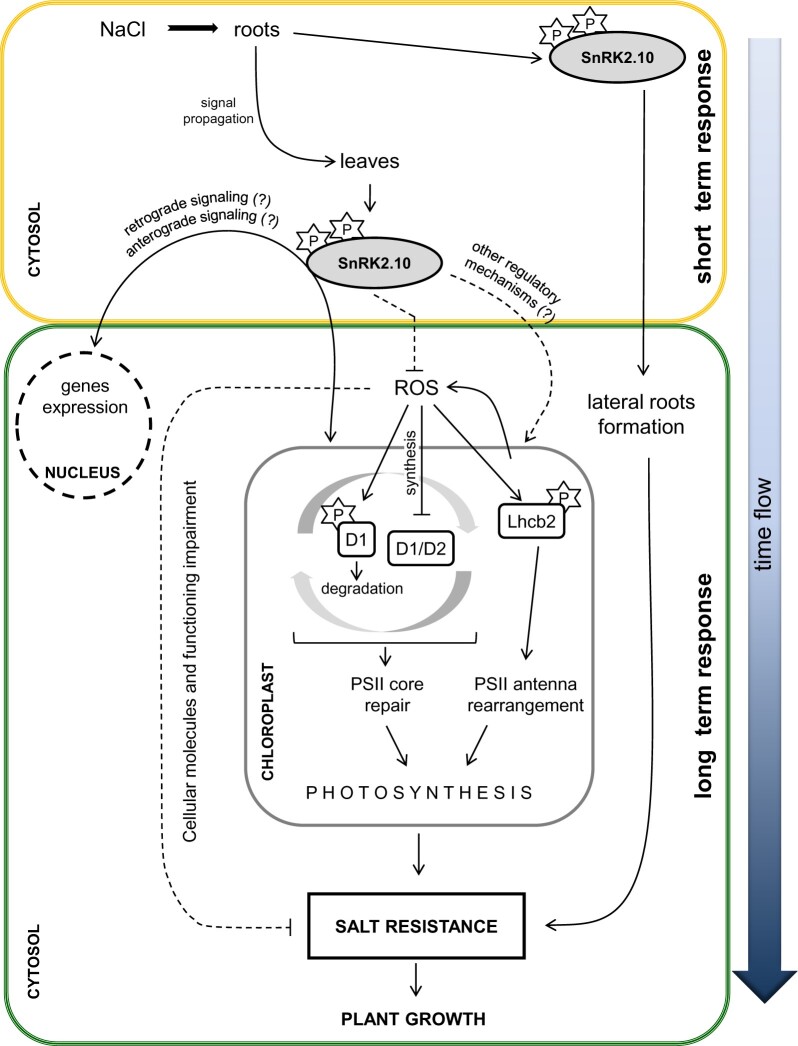
Model presenting postulated role of SnRK2.10 in *A. thaliana* response to salinity. Model is based on the results of McLoughlin et al. (2012) and Szymańska et al. (2019) and the present data. In response to salinity, SnRK2.10 is rapidly activated in plant roots and leaves. SnRK2.10-dependent signaling during early response to salinity is required for an appropriate regulation of stress-genes expression, maintenance of ROS accumulation, and also chloroplast–nucleus–chloroplast signaling in later stages of the response. Thus, SnRK2.10 has an impact on cell functioning, for example, photosynthesis, and as a consequence, influences plant susceptibility to salinity.

Further analysis of other photosynthesis-related proteins, like RbcL, RbcS, PsbP, PsbO, and AtpB, revealed that in the conditions applied only RbcL was transiently accumulated under salinity (between 6 h and 3 d of stress) and then its level dropped at Day 6 (Supplemental Figure S10). There were no differences observed between the lines studied. In parallel, the expression of selected genes coding for proteins involved in photosynthesis maintenance was evaluated by RT-qPCR (Supplemental Figure S11). Their expression was downregulated under salinity but no significant differences were found between the wt plants and the *snrk2.10* mutants. In contrast, expression of *BAP1* (BON association protein 1) and *DREB19* (dehydration response element-binding protein 19), two stress-responsive genes (Yang et al., 2007; Krishnaswamy et al., 2011) was strongly enhanced under salinity (Supplemental Figure S11). Detected induction of expression was markedly lower in the *snrk2.10* mutants than in the wt plants.

### SnRK2.10 influences ROS accumulation under salinity stress

To investigate why the extent of D1 protein phosphorylation is elevated and its level reduced in the *snrk2.10* mutant, we monitored ROS accumulation in leaves of salt-treated mutants and wt plants. It has been reported that prior to its phosphorylation and degradation, D1 protein undergoes oxidative damage caused by diverse ROS, including superoxide anion (O_2_^.−^), hydrogen peroxide (H_2_O_2_), and hydroxyl radical (HO^•^; Kale et al., 2017). It was also proposed that ROS are the main factors that initiate the D1 protein degradation. Salinity is known to trigger the production of ROS that may affect chloroplast functioning (Murata et al., 2007). For that reason, we visualized the accumulation of H_2_O_2_ in Arabidopsis leaves of salt-treated and not-treated plants by DAB (3,3′-diaminobenzidine) staining. Hydrogen peroxide was selected for detection as a representative ROS molecule due to its relatively long life and high mobility. Corresponding leaves were collected for staining from wt plants and *snrk2.10* mutants before and after plant exposition to salinity stress. The *snrk2.10* leaves were slightly more intensely stained than wt leaves already after 1 d of salinity treatment and the difference in coloration became more pronounced with stress duration (Figure 8, A). Furthermore, we analyzed the level of H_2_O_2_ by the Amplex Red method in leaves of control plants and leaves of plants treated for 6 d with NaCl. Absorbance (560 nm) detected in samples from *snrk2.10* leaves after salinity was significantly higher than from wt plants (Figure 8, B). Obtained results indicate higher H_2_O_2_ accumulation in the *snrk2.10* mutant plants under salinity stress. In contrast, in *snrk2.4* leaves, the level of H_2_O_2_ after 6 d of salinity was similar to that in wt plants (Supplemental Figure S12).

## Discussion

Plant response to salinity comprises two major phases. The first one is salt sensing and signal propagation by a network of protein kinase/phosphatase cascades with an involvement of secondary messengers (like ROS, reactive nitrogen species [RNS], Ca^2+^). This phase is responsible for a fast response to water deficit and ion imbalance. The second phase is related to the cytotoxic effects of ion accumulation (Isayenkov and Maathuis, 2019). The activation of SnRK2.4 and SnRK2.10 observed by us belongs to the fast-response events. It has been shown that under salinity these enzymes are activated in Arabidopsis roots within seconds (McLoughlin et al., 2012). In our study, both SnRK2s were activated in Arabidopsis leaves within only 5 min after the roots had been exposed to salinity. This activation occurred most probably via a fast salt stress signaling system transferring the information about salinity from plant roots to leaves. It has been proposed that the signal propagation system mainly involves waves of ROS production and of calcium release, Na^+^ transport, and/or loss of cellular turgor progressing across plant organs (Choi et al., 2016; Mitller, 2017). The phosphorylation of SnRK2.10 as an element of early response to salinity and H_2_O_2_ has been confirmed by a large-scale phosphoproteomic study (Chen and Hoehenwarter, 2015). Previously we have also reported that NtOSAK (*Nicotiana tabacum* osmotic stress-activated protein kinase), a homolog of Arabidopsis SnRK2.10 and SnRK2.4, is activated in response to salinity or hyperosmotic stress in NO-dependent manner, and also by application of H_2_O_2_ or a NO donor (Lamotte et al., 2006; Wawer et al., 2010). Thus, it seems likely that the NaCl-induced activation of the SnRK2.4/2.10 kinases in Arabidopsis leaves occurs, among others, due to ROS formation. On the other hand, it has been shown that the ABA not-activated SnRK2s take part in ROS production/accumulation under stress conditions evoked by salinity, high osmotic pressure, or cadmium ions (Kulik et al., 2012; Szymańska et al., 2019). According to Szymańska et al. (2019), SnRK2.4 and SnRK2.10 positively regulate ROS accumulation at early stages of the plant response to salinity. Thus, one can hypothesize that SnRK2s could be involved in a positive feedback loop, where the SnRK2-dependent ROS production in roots initiates a root—rosette ROS wave leading to ROS production in the upper parts of the plant activating SnRK2s in leaves (as presented in Figure 9). However, also other mechanisms of SnRK2.4/2.10 activation in Arabidopsis leaves should be taken into account because, despite intense studies, details of the signaling pathways and mechanisms leading to the activation of group 1 SnRK2 kinases are not yet fully understood. Recently, it has been revealed that three B4 Raf-like MAP kinase kinase kinases (RAF18/20/2) phosphorylate and activate group 1 SnRK2s under osmotic stress (Soma et al., 2020). Whether these kinases are activated in ROS-dependent manner is not known.

The analysis of various chlorophyll fluorescence parameters is a fast and informative way of estimating the changes in the performance of photosynthetic light reactions in plants challenged with various environmental stresses. Saline conditions lead to a downregulation of photochemical and upregulation of non-photochemical processes (Acosta-Motos et al., 2017), but the extent of such changes depends on, for example, salt concentration and growth conditions. In our experimental model, 100 mM NaCl did not significantly affect the photosynthetic performance of wt plants, while in the *snrk2.10* mutants, we observed a transient intensification of non-photochemical processes; which returned to the initial values by the end of the experiment indicating activation of acclimation mechanisms. At 150 mM NaCl treatment, which produced a more pronounced response, in all the lines examined, we observed similar effects of salt treatment (1) a decrease of content of chlorophyll and carotenoids as well as the Chl *a/b* ratio; (2) a decrease of the photochemical quantum yield of PSII and PSI and the photochemical quenching parameter qL; (3) an increase of the NPQ parameter and the de-epoxidation status of xanthophylls. These results are in line with other studies on the effects of saline stress and water deficit on photosynthesis (Chen et al., 2016, 2017; Yi et al., 2018; Tsai et. al., 2019; Grieco et al., 2020; Yang et al., 2020; see also review by Acosta-Motos et al., 2017). Interestingly, in the *snrk2.10* lines, the changes appeared earlier than in wt plants indicating that the excitonic pressure-inducing mechanisms dissipating excess of absorbed light energy was higher in those lines. We found that both, SnRK2.4 and SnRK2.10, limited the growth retardation of rosettes caused by a prolonged salt stress. However, unlike *snrk2.10*, the *snrk2.4* mutants subjected to salinity showed no significant differences in photosynthesis and ROS accumulation compared to wt plants. This suggests that both kinases may enhance the plant tolerance to salinity, but by different mechanisms. The mechanisms controlled by SnRK2.4 in response to salinity require further studies.

To fathom the involvement of SnRK2.10 in the regulation of photosynthesis in plants challenged by NaCl, we monitored the expression of photosynthesis-related genes and level of relevant proteins accumulation. SnRK2.10 did not affect the genes expression but the level of Lhcb2, D1, and D2 proteins and their phosphorylation status were affected in *snrk2.10* knockout mutants. Both PSII core proteins (D1 and D2) rapidly undergo oxidative damage and degradation (Gururani et al., 2015; Liu et al., 2019), therefore we postulate that under salinity SnRK2.10-dependent signaling pathways limit ROS accumulation thereby preventing oxidative damage of D1 and D2. One should note that, according to Murata et al. (2007), ROS may also increase the extent of photoinhibition by suppressing D1 protein translation and consequently inhibiting the repair of PSII. A lack of SnRK2.10 also enhanced the level of Lhcb2 phosphorylation under salinity conditions. Lhcb2 is the most abundant protein of PSII antenna light-harvesting complexes which undergoes controlled partial phosphorylation under optimal growth conditions and enhanced phosphorylation under stress. This phosphorylation is required for relocation of Lhcb2 and other Lhcb proteins from PSII to PSI antenna supercomplexes which prevents a pronounced imbalance in the photosynthetic electron flow between PSII and PSI and stimulation of redox signaling pathways (Gururani et al., 2015). It is also known that the phosphorylation of PSII core and antenna proteins increases thylakoid membrane fluidity (Pesaresi et al., 2011). Our results showed that under NaCl stress, the Lut/β-car ratio is stable, and thus optimal membrane fluidity is maintained despite increased protein phosphorylation. Taken together, our results indicate that SnRK2.10 is required to ensure a correct turnover of PSII core proteins and rearrangement of antenna complexes under salinity conditions, which has an impact on the photosynthesis efficiency and growth potential of plants, as proposed in Figure 9.

PSII core proteins are known to be phosphorylated mainly by the STN8 kinase, whereas the LHCII proteins are phosphorylated by STN7 (Bonardi et al., 2005; Pesaresi et al., 2011; Longoni et al., 2015). A lack of SnRK2.10 had no impact on the accumulation of *STN7 and STN8* transcripts or on the STN7 protein content, therefore the question of how SnRK2.10 may affect STN7 and STN8 activity remains open. The mechanisms of activation of these kinases are still poorly understood. It has been established that STN7 is activated upon salinity (Chen and Hoehenwarter, 2015) and in redox-dependent manner through its interaction with the cytochrome b_6_f complex upon reduction of the plastoquinone pool (for review see Grieco et al., 2016), so it is possible that SnRK2.10 regulates STN7 indirectly through its influence on ROS accumulation and redox homeostasis. It has been also suggested that STN7 and STN8 have partially overlapping substrate ranges and do not affect each other’s accumulation (Pesaresi et al., 2011; Wunder et al., 2013).

A putative phosphatase dephosphorylating STN8 substrates from PSII core has been identified as a chloroplast class PP2C phosphatase (PBCP; Samol et al., 2012). Dephosphorylation of PSII core proteins is crucial for an effective degradation of D1 (Gururani et al., 2015). Another PP2C-type protein phosphatase called thylakoid-associated phosphatase 38/protein phosphatase 1 (TAP38/PPH1) was identified as a negative regulator of both STN7 and STN8 kinases and is supposed to be constitutively expressed and active, and thus redox-independent (Pribil et al., 2010). It is very unlikely that STN7, STN8, PBCP, or TAP38 interact with SnRK2.10 directly because the kinase has never been detected in the chloroplast. It has been shown recently that cytosolic OST1 (SnRK2.6) kinase interacts with and phosphorylates chloroplast PsbP domain protein 5 (PPD5) (Hong et al., 2020). PPD5 negatively regulates drought resistance by modulating guard cell H_2_O_2_ accumulation *via* an SnRK2.6-dependent pathway. Because both proteins interact near or around the chloroplast, it has been suggested that phosphorylation may occur in the cytoplasmic side of the chloroplast membrane or PPD5 is phosphorylated before being transported to the chloroplast.

It has been shown that phosphorylation of chloroplast-designated preproteins by cytosolic kinases is one of the key steps ensuring their sorting and specific targeting to this organelle during plastid maturation and stress response in Arabidopsis and pea (Waegmann and Soll, 1996; Martin et al., 2006; Lamberti et al., 2011b; Zufferey et al., 2017; Eisa et al., 2019; Hristou et al., 2020). The serine/threonine/tyrosine kinase 8 (STY8), STY17, and STY46 kinases phosphorylate chloroplast preprotein transit peptides ensuring their binding of a 14–3–3 dimer, which enhances association to the TOC receptor located at the outer envelope membrane of the chloroplast (Martin et al., 2006; Lamberti et al., 2011a, 2011b). Identification of SnRK2.10-interacting proteins coupled with phosphoproteomic studies seems to be the next big challenge towards understanding the regulatory role of SnRK2.10 in chloroplast functioning and chloroplast protein/preprotein phosphorylation.

Some evidences for SnRKs involvement in maintenance of chloroplast proteins phosphorylation have been already delivered. Nakarinen et al. (2016) found that SnRK1 (for review see Jamsheer et al., 2021) alters the phosphorylation status of several plastid and mitochondrial proteins, for example, PsaP, LHCB4.2, PTF1, PTAC5, and CP29. SnRK1 is a master metabolic regulator of plant growth under energy deprivation conditions and localizes mainly to the cytoplasm and nucleus, but both its subunits, AKIN10 and AKIN11, were also ambiguously seen in the chloroplast (Fragoso et al., 2009). Wurzinger et al. (2018) suggested that TFs directly phosphorylated by SnRK1 (e.g. bZIP63; Mair et al., 2015) are predicted to bind to nuclear-encoded plastid and mitochondria genes, possibly altering their transcription. According to this suggestion, SnRK1 may take part in an anterograde (nucleus to organelle) signaling which is based on the delivery of preproteins to the organelle (Kmiecik et al., 2016; Wurzinger et al., 2018). This theory is also supported by phosphoproteomic data showing that SnRK1, SnRK2.4, and SnRK2.6 may phosphorylate TOC159 protein (Wang et al., 2013; Nakarinen et al., 2016; Wang et al., 2020a), which is a component of TOC–TIC import machinery which provides recognition and translocation of the preproteins at the plastid envelope (Demarsy et al., 2014). It has been proposed that phosphorylation of A-domain, for instance by the regulatory kinase at the outer chloroplast membrane 1 (KOC1), probably modulates interactions of TOC159 with other TOC components and with specific sets of client preproteins (Zufferey et al., 2017). However, further detailed study on TOC159 phosphorylation by SnRK2s *in planta* is required.

The question of whether SnRK2.10 may be involved in retrograde signaling stays open for an investigation. Several types of molecules were recognized as possible retrograde signals so far, among others sugars, calcium, transcription factors, hormones, and protein kinases (CDPKs, MPK3/6), but it seems that one of the most important are ROS (Guo et al., 2016; for review see Stael et al., 2015; Kmiecik et al., 2016; Wang et al., 2020b). For instance, MAPK pathways are central regulators of plant response to diverse environmental stimulus, which stay under the control and control by itself cellular ROS and redox homeostasis and thus take part in the retrograde signaling (for review see Dietz et al., 2016; Liu and He, 2017). It has been proposed that salinity induces the formation and accumulation of ROS in plants that occur in two phases. The ROS produced at the very early stages of the stress response act as signaling molecules activating defense mechanisms, while those produced in an uncontrolled manner at later stages are detrimental to the plant by damaging diverse essential molecules and affecting biochemical processes, including photosynthesis (Mittler, 2017). Dietz et al. (2016) and Stael et al. (2015) report that ROS (including chloroplast-delivered H_2_O_2_) and calcium participate in early retrograde signaling triggered by abiotic and biotic stimulus. Moreover, signals initiated by ROS accumulating in the cytoplasm may be perceived by chloroplasts and in a feedback loop play a role in retrograde signaling to the nucleus (Stael et al., 2015). According to Szymańska et al. (2019), in *snrk2.10* leaves ROS production at the early response to salinity is disturbed. Thus, we hypothesize that the enhanced ROS accumulation in *snrk2.10* plants challenged with long-term salinity could be due to disturbances in the initial phase of ROS production, chloroplast to nucleus communication, signaling and proper initiation of defense mechanisms. We propose that the elevated ROS accumulation may exacerbate the impairment of photosynthesis and growth retardation, bringing about an overall increased susceptibility of *snrk2.10* mutants to salinity.

In our study, we found two genes which expression was altered in *snrk2.10* plants under salinity, *DREB19*, and *BAP1*. *DREB19* encodes a member of ERF/AP2 transcription factor family. This transcription factor is a positive regulator of plant tolerance to drought, strongly induced upon drought and salinity and by chloroplast ROS formation caused by paraquat treatment (Krishnaswamy et al., 2011; Mehterov et al., 2012; Sujeeth et al., 2020). BAP1 is known as a suppressor of programmed cell death inducted by cold and paraquat (Yang et al., 2007; Van Buer et al., 2016). Expression of *BAP1* is controlled by EXECUTER2-mediated chloroplast to nucleus ROS signaling so it is known as a chloroplast ROS signaling marker gene (Lee et al., 2007; Van Buer et al., 2016). We hypothesize that markedly lower induction of *DREB19 and BAP1* in *snrk2.10* mutants may drive an impaired salinity response in those plants. Lower expression of these genes with a simultaneous higher accumulation of ROS in *snrk2.10* mutants may indicate, inter alia, a disturbed transmission of the oxidative stress signal to the nucleus. This hypothesis and detailed gene expression analysis in *snrk2.10* lines need further investigation to put more light on the putative involvement of SnRK2.10 in ROS signaling.

In summary, we have shown that the SnRK2.10 kinase is involved in mitigating the susceptibility of *A. thaliana* plants to salinity and indicated a possible mechanism of this regulation. We found that SnRK2.10 is rapidly activated in leaves in response to NaCl. During the growth in a salt-containing medium for several days, *snrk2.10* mutants reacted more severely than wt plants did, showing lower maximal quantum yield of PSII, higher NPQ, and reduced adaptation of the photosynthetic apparatus to an increasing light intensity. We found a higher ROS level in the *snrk2.10* mutants than in wt plants, likely interfering with the reconstruction and rearrangement of damaged core and antenna protein complexes in PSII. Based on the present findings and published data, we propose a model showing the role of SnRK2.10 in the response of plants to salinity (Figure 9).

## Materials and methods

### Plant lines, growth conditions, and stress application

Several *Arabidopsis thaliana* lines were used: wt Col-0, *snrk2.10-1* (WiscDsLox233E9), *snrk2.10-3* (SAIL_698_105), *snrk2.4-1* (SALK_080588), *snrk2.4-2* (SALK_146522), *stn7* (SALK_07325), and *stn8-1* (SALK_060869). Lines expressing SnRK2.4-GFP, SnRK2.10-GFP, pSnRK2.4:SnRK2.4-YFP, and pSnRK2.10:SnRK2.10-YFP were kindly provided by Prof. Christa Testerink, the Wageningen University.

Plants were grown under short-day conditions (8 h light 23°C/16 h dark 21°C) in a hydroponic culture (Araponics system) in media previously described in Kulik et al. (2012). Five-week-old plants were treated or not (control) with 100 mM NaCl in growth medium for up to 9 d or 150 mM NaCl or 150 g/L PEG 8000 for up to 6 d.

For growth assay, rosettes were collected and immediately weighed before and after 6 d of plant exposure to 150 mM NaCl. After drying overnight at 70°C, the rosettes were weighed again.

### Chlorophyll *a* fluorescence imaging

Chlorophyll fluorescence images of rosettes were recorded using FluorCam FC 800-C (Photon System Instruments, Brno, Czech Republic) with a 512 × 512 pixels resolution. Before measurements plants were dark-adapted for 30 min and after that *F*_0_ and *F*_M_ values were determined as described previously (Mazur et al., 2016). The maximal quantum efficiency of PSII (*F*_V_/*F*_M_) was calculated from the formula (*F*_M_ – *F*_0_)/*F*_M_ using FluorCam v7.0 software.

### Chlorophyll *a* fluorescence and P700 measurements

Measurements were carried out using a Dual-PAM-100 (Heinz Walz GmbH, Effeltrich, Germany) pulse amplitude modulation fluorometer—and the automated Induction and Light Curve routine provided by the DualPam software, as described previously (Mazur et al., 2016). Briefly, plants were dark-adapted for 30 min before the measurements and after determination of *F*_0_, *F*_M_, and *P*_M_, the actinic light (75 μmol photons s^−1^ m^−2^) was turned on and *F*_M_′ and *P*_M_′ values were determinated. Next, a light curve was obtained by illuminating light-adapted leaves with exponentially increasing actinic light intensity (from 5 to 5,146 μmol photons s^−1^ m^−2^). Photosynthetic parameters were calculated according to the formulae specified previously (Mazur et al., 2016).

### 77 K steady-state fluorescence

Leaf samples were ground in chilled 20 mM Hepes-NaOH (pH 7.5) buffer containing 330 mM sorbitol, 15 mM NaCl and 4 mM MgCl_2_. Homogenates were filtered through 100-µm nylon mesh, diluted to chlorophyll concentration of 10 µg mL^−1^, and placed in a polytetrafluoroethylene cuvette. Low temperature (77 K) fluorescence emission spectra were recorded using a modified Shimadzu RF-5301PC spectrofluorimeter as described previously (Mazur et al., 2016). Excitation wavelength was set at 440 nm, excitation and emission slits at 5 nm, and emission scans were taken in the range of 600–800 nm through an LP600 filter.

### RT-qPCR

Rosettes were ground to fine powder in liquid nitrogen. RNA was extracted with Trizol (Molecular Research Center, Cincinnati, OH, USA) reagent according to the manufacturer’s instructions and treated with DNase 1 (Thermo Scientific, Waltham, MA, USA). Reverse transcription was performed on 1 µg of RNA using the RevertAid First Strand cDNA Synthesis Kit (Thermo Scientific). The resulting cDNA was diluted 10-fold with water and 1 μL of the sample was assayed by qPCR in a Step One Plus device (Applied Biosystems) using GoTaq^®^ qPCR Master Mix (Promega) and specific pairs of primers (Supplemental Table S1). Expression levels were calculated relative to the housekeeping genes *PDF2* (*At1g13320*) and *PEX4* (*At5g25760*) using the delta-delta Ct method.

### Preparation of protein extracts

Protein extracts from rosettes before and after treatment with 150 mM NaCl were prepared as previously described by Kulik et al. (2012) or the method described by Rudowska et al. (2012) was applied for immunological detection of chloroplast proteins.

### Immunoblotting

Immunoblotting was performed as previously described by Kulik et al. (2012). A set of different proteins was detected. For detection of GFP-conjugated proteins, HRP-conjugated anti-GFP antibodies (Santa Cruz Biotechnology, Dallas, TX, USA) diluted 1:1,000 were used following manufacturer's protocol. Antibodies recognizing D1, phosphorylated D1, D2, Lhcb2, phosphorylated Lhcb2, STN7, RbcL, RbcS, PsbP, PsbO, and AtpB were obtained from Agrisera and used according to manufacturer's recommendation. Membrane stripping was performed using Re-blot Plus Strong Antibody Stripping Solution (EMD Millipore) and the same membranes were used for detection of various proteins.

### Immunoprecipitation and immunocomplex kinase activity assay

For immunoprecipitation of GFP-conjugated proteins GFP-Trap_A (Chromotek) resin was used as described in Supplemental Methods and Results. Immunoprecipitation using anti-SnRK2.4/SnRK2.10 antibodies and immunocomplex kinase activity assay were performed as described in Kulik et al. (2012).

### In-gel kinase activity assay

In-gel kinase activity assays were performed as described in Kulik et al. (2012).

### H_2_O_2_ detection

Hydrogen peroxide accumulation in leaves was estimated by staining with 3,3′-diaminobenzidine (DAB) as described by Daudi and O’Brien (2012) and by Amplex Red method according to Guo et al. (2017).

### Statistical analysis

The results are presented as arithmetic means with standard deviations. Statistical significance of differences between groups was tested by Student’s *t*-test or ANOVA and post hoc Tukey’s test (*P* = 0.05).

Immunoprecipitation and immunocomplex kinase activity assay, pigments extraction and analysis, localization of SnRK2.4 and SnRK2.10, stomatal conductance measurements, stomatal index calculation, and chlorophyll *a* fluorescence imaging methods are provided in the Supplemental Methods section.

### Accession numbers

Gene accession numbers used in this study: SnRK2.4 (AT1G10940), SnRK2.10 (AT1G60940), D1/PSBA (ATCG00020).

## Supplemental Data

The following materials are available in the online version of this article.

**Supplemental Methods**.

**Supplemental Results**.

**Supplemental References**.

**Supplemental Figure S1.** Stomatal conductance and stomatal index in wt and *snrk2.10* mutant lines.

**Supplemental Figure S2.** *SnRK2.4* and *SnRK2.10* expression under salt stress.

**Supplemental Figure S3 *.*** Localization of SnRK2.4 and SnRK2.10 in the leaf tissue.

**Supplemental Figure S4.** Effect of 100 mM NaCl on chlorophyll *a* fluorescence distribution of wt and *snrk2.10* mutant plants.

**Supplemental Figure S5.** Effect of 150 mM NaCl and 150 g/L PEG 8000 on efficiency of photosynthetic light reactions of wt and *snrk2.10* mutant plants under steady light intensity.

**Supplemental Figure S6.** Effect of 100 mM NaCl on efficiency of photosynthetic light reactions of wt and *snrk2.10* mutant plants under steady light intensity.

**Supplemental Figure S7.** Effect of salt stress on efficiency of photosynthetic light reactions of wt and *snrk2.4* mutant plants.

**Supplemental Figure S8.** Effect of salt stress on chlorophyll and carotenoid composition in wt and *snrk2.10* plants.

**Supplemental Figure S9.** STN7 and STN8 affect proteins levels and their phosphorylation status in plant chloroplasts under salt stress.

**Supplemental Figure S10.** Levels of photosynthesis-related proteins in wt, *snrk2.10-3*, and *snrk2.10-1* plant lines under long-term salinity.

**Supplemental Figure S11.** Expression of photosynthesis- and stress-related genes under salinity.

**Supplemental Figure S12.** Accumulation of H_2_O_2_ in *A. thaliana* leaves of *snrk2.4* mutant plants under long-term salinity.

**Supplemental Table S1.** List of primers used in this study.

## Supplementary Material

kiab438_Supplementary_Data

## Notes

The SNF1-Related protein Kinase 2.10 (SnRK2.10) improves plant tolerance to salinity by maintaining ROS accumulation and protecting the photosynthetic machinery.

A.K. designed and supervised the study; A.K., R.M., M.G., and G.D. designed and supervised experiments and analyzed data; A.Z. performed preliminary experiments; A.K., R.M., J.M., A.A.-M., M.G., L.P-K., and A.C. performed the experiments; A.K., G.D., R.M., and M.G. interpreted results and wrote or revised the manuscript; A.K. supervised the writing and edited the final version of the manuscript.

The author responsible for distribution of materials integral to the findings presented in this article in accordance with the policy described in the Instructions for Authors (https://academic.oup.com/plphys/pages/general-instructions) is: Anna Kulik (anja@ibb.waw.pl).
