# Supplementary material for: The SnRK2.10 kinase mitigates the adverse effects of salinity by protecting photosynthetic machinery
Source: Plant Physiol. 2021 Sep 23;187(4):2785–802. doi: 10.1093/plphys/kiab438 (PMC8644180; doi:10.1093/plphys/kiab438)
Supplement: kiab438_Supplementary_Data [file kiab438_supplementary_data.pdf]

Supplemental Methods

Immunoprecipitation and Immunocomplex Kinase Activity Assay

For immunoprecipitation of GFP-fused proteins 200 µg of crude protein extract from rosettes of plants treated with 250 mM NaCl for up to 6h was incubated with 10 µL of GFP-Trap®_A (Chromotek) for 2.5h with gentle rocking. After intensive washing agarose beads with bound immunocomplexes were suspended in 20 mM Tris–HCl, pH 7.5 supplemented with 150 mM NaCl and 4 µg of Myelin Basic Protein (Sigma-Aldrich) per sample. To each sample ATP supplemented with 1 µCi of [γ-^{32}P]ATP in kinase buffer (25 mM Tris-HCl, pH 7.5, 5 mM EGTA, 1 mM DTT, 30 mM $MgCl_2$) was added to 50 µM final concentration. After 15 min. of incubation at 37°C samples were mixed with Laemmli sample buffer and incubated for 3 min. at 95°C with vigorous shaking. Proteins were separated on 12% SDS polyacrylamide gel and signal detected on Medical X-ray Blue/MXBE Film (Carestream).

Pigments extraction and analysis

Chlorophylls and carotenoids were quantitated by spectroscopic measurements following extraction with 80% acetone from powdered leaves according to Lichtenthaler (1987). For detailed analysis by UPLC, a multi-step extraction procedure was applied as described in Szalonek et al. (2015).

Extracted pigments were separated using an Acquity Ultra Performance LC system (Waters) and an Acquity UPLC HSS T3 (1.8 µm, 1.0 × 150 mm) analytical column. Pigments were eluted with a step gradient of solvent A (water : methanol 15:85, v/v) and B (methanol : 2-propanol : hexane 2:1:1, v/v) as described previously (Skupien et al., 2017). For quantification of carotenoids, α-tocopherol, and plastoquinone chromatograms at 436, 291, and 255 nm, respectively, were integrated using MassLynx 3.5 software (Waters) and results were presented as mol% or mol/mol ratio based on calibration curves obtained for appropriate standards.

Localization of SnRK2.4 and SnRK2.10

Transgenic plants expressing YFP under the native promoter (pSnRK2.4::SnRK2.4-YFP and pSnRK2.10::SnRK2.10-YFP, McLouglin et al., 2012) were used to study the localization of SnRK2.4 and SnRK2.10 kinases in photosynthetic tissue. Plants were grown as described in McLouglin et al., 2012. Leaves from three to four weeks-old plants were detached, infiltrated with water and mounted on slides with homemade chambers. Imaging of YFP fluorophore was carried out on an inverted microscope (Nikon, TE2000) with a confocal laser-scaning mode EZ-C1. YFP fluorescence was excited with blue light emitted by a Sapphire 488 nm

laser (Coherent, USA) set at 30% then collected with a 525/40 nm emission filter and displayed in false green. The gain of the photomultiplier was set to 8.9. Since the collected signal was very weak, a wider pinhole size (100 μm) was used. Simultaneously the chlorophyll autofluorescence was detected by 610 long pass filter and displayed in false magenta. The gain of the photomultiplier was set to range 6.3-6.9. Palisade mesophyll has been imaged through the epidermis layer using 60x oil immersion objective (Nikon, CFI Plan Apochromat NA 1.4). Single confocal sections and stacks were collected for both lines. The images were digitally processed using FIJI software (NIH, Bethesda, MD, USA) and the figures compiled in Adobe Photoshop 6.0 38 CE (Adobe Systems Inc.).

Stomatal conductance measurements

Six-weeks old plants of wild type and *snrk2.10* insertion mutants were grown in hydroponic culture and treated with control media or media supplemented with 150 mM NaCl for up to six days. Stomatal conductance / stomatal aperture in terms of leaf conductance to water vapour was measured with AP4 Leaf Porometer (DeltaT Devices) on fully developed leaves at similar age and expressed in mmol $H_2O/m^2/s$. For each biological replication three leaves from five plants (n=15) per genotype and treatment were analyzed.

Stomatal index calculation

The 5th and 6th leaves were detached from 4-weeks-old plants grown in soil. 0,5cm^2 pieces were cut from the middle leaf blade away from the leaf veins. Pieces from the adaxial and abaxial sides of the leaf were attached to the adhesive tape and the top surface of the tape was immediately covered with a quick-drying nail polish. After 20 min of drying, the top surface was removed and mounted onto a slide. Microscope images of the peeled surface area were taken in several contiguous but not overlapping areas of 0,498 mm^{-2} (10x objective, Nikon, Eclipse E-800), recorded by a monochromatic camera (ORCA ER, Hamamatsu) and counted in FIJI software (https://imagej.net/contribute/citing, NIH, Bethesda, MD, USA, https://www.ncbi.nlm.nih.gov/pmc/articles/PMC3855844/). The total number of images collected and counted was 100 for each line. Stomatal and other epidermal cell densities were counted as a number of cells per area unit (mm^2). The stomatal index (SI) was calculated as the percentage of the stomata to all epidermal cells, according to the formula: SI = (number of stomata) / (number of total epidermal cells) x100%.

Chlorophyll *a* fluorescence imaging

Chlorophyll *a* fluorescence images were recorded using the Maxi version of Imaging-PAM chlorophyll fluorescence system (Heinz Walz, Germany). The *A. thaliana* plants grown and treated with salt containing or not containing media were dark-adapted for at least 25 min,

measurements were performed and plants were returned to the phytotron. The measurements were performed for seven days.

The fluorescence images were recorded with a resolution of 640 × 480 pixels and with the camera parameters set to avoid pixel saturation. Minimal (F_0) fluorescence was determined using weak blue modulating light of 0.5 μmol photons m^{-2} s^{-1}, whereas maximal (F_M) fluorescence was measured through 0.84 s of saturation blue light pulse with 2,700 μmol photons m^{-2} s^{-1}. Next, the blue actinic light of 54 μmol photons m^{-2} s^{-1} was on, and saturation pulses at 20 s time intervals during 240 s were applied and F_M' values were measured. The recorded data were analyzed using ImagingWinGigE software and photosynthetic parameters: F_V/F_M, Y(II), NPQ, NPQ_{max}, and qL for 5th - 7th leaves were calculated. For each leaf, the data were normalized to 1 on day 0 of the control, NaCl, or PEG treatment.

Supplemental Results

A lack of SnRK2.10 kinase does not affect the changes in abundance and composition of chlorophylls and carotenoid pigments in plants subjected to salinity

Carotenoids are key components of the xanthophyll cycle responsible for protection of unsaturated fatty acids of chloroplast lipids from stress caused by photooxidation (Foyer and Shigeoka, 2011; Horton, 2012; Ashraf and Harris, 2013). A total content of chlorophylls and carotenoids as well as the Chl *a/b* ratio under stress conditions usually results from the decomposition of chlorophyll-protein complexes. In *A. thaliana* plants exposed to 150 mM NaCl the content of chlorophylls and carotenoids in dry tissue decreased linearly from day one of exposure, similarly in *wt* and *snrk2.10* plants (Fig. S8 A, B). Also the Chl *a/b* ratio decreased linearly during the salt stress, but in the *snrk2.10* lines this decrease was apparent sooner than in the wt (Fig. S8 C). The changes in chlorophylls/carotenoids ratio in both mutant lines were less visible than in wt and fluctuating around the value of 5.5 both in control and salt-treated plants (Fig. S8 D).

Carotenoids are associated with chlorophyll-protein complexes where they play a significant role in the dissipation of an excess of light energy. Furthermore, together with other lipid-soluble molecules, such as plastoquinones (PQ) and α-tocopherols (α-T), carotenoids are localized directly in the lipid bilayer and modify the fluidity of thylakoid membranes as well as act as antioxidants preventing lipid peroxidation. Therefore, a detailed analysis of these molecules by UPLC might provide information related to the response to salinity stress. The wt and mutant lines showed different profiles of accumulation of specific carotenoids during growth in control conditions (Fig. S8 E-H). In wt plants, a significant increase in the abundance of neoxanthin (Neo) was associated with a stable level of violaxanthin (Vio), a slow decrease of lutein (Lut), and β-Carotene (β-Car) level. In the *snrk2.10* plants grown in control conditions only β-Car showed a decline (in the *snrk2.10-3* line only), Neo, Lut, Vio, being stable (or, in the *snrk2.10-1* line, showing transient fluctuations). The content of other carotenoids localized in thylakoids: zeaxanthin, antheraxanthin, and cryptoxanthin did not exceed 0.5 mol% in all lines (not shown). The de-epoxidation state of xanthophylls (DEPS) in control conditions was relatively low and similar in all lines, indicating the same capacity to dissipate excessive light energy by the xanthophylls-dependent processes (Fig. S8 I). The Lut/β-Car ratio, which is a good estimator of thylakoid membrane fluidity, was increasing during growth in control conditions all examined lines (Fig. S8 J), suggesting an increase of the rigidity of thylakoid membranes with plant age. The relative content of the sum of PQ and α-T slightly decreased during the growth of wt plants in control conditions and was stable in both mutant lines (Fig. S8 K). At the end of cultivation in control conditions the levels of β-

Car, PQ, and α-T were similar in all examined lines indicating a similar 'antioxidative potential'. In plants exposed to 150 mM NaCl the content of Neo increased during the time and was higher comparing to control conditions except for the wt plants (Fig. S8 E). In contrast, the Lut level decreased in time during salinity and was lower than in control except for the wt plants (Fig. S8 G). The level of β-Car in all NaCl-treated lines showed similar changes as in control conditions, (Fig. S8 H). However, the Lut/β-Car ratio was unchanged in wt and *snrk2.10-3* lines and noticeably decreased in *snrk2.10-1* plants at the end of salt treatment comparing to the control conditions (Fig. S8 J). An increase of DEPS was observed in all lines, but was slightly delayed in the mutant lines; at the end of salt treatment, the DEPS values were 3-4 times higher than in control conditions (Fig. S8 I). Similarly, the content of membrane antioxidants PQ and α-T increased rapidly in all examined lines and was two times higher than in the control conditions (Fig. S8 K).

To sum up, the response of the wt and mutant lines to NaCl treatment was similar and included (i) a decrease of membrane fluidity, (ii) an increase of the membrane 'antioxidant potential', and (iii) an increase of the de-epoxidation state of xanthophylls.

Phosphorylation of LHCII proteins affects phosphorylation of D1 protein under salinity

It has been established that the phosphorylation of PSII and LHCII proteins in Arabidopsis is carried out by two state transition kinases, STN8 (State Transition 8) and STN7 (State Transition 7), respectively. STN8 plays a role in the modulation of the thylakoid ultrastructure phosphorylating mainly PSII core proteins, whereas STN7 is responsible for the maintenance of the redox status of the photosynthetic electron transport chain and counteracts the imbalances in energy distribution between PSII and PSI under unfavorable environmental conditions phosphorylating, among others, the Lhcb proteins (Bonardi et al., 2005; Pesaresi et al., 2011; Longoni et al., 2015). To determine whether the two STN kinases also reciprocally affect the phosphorylation status of each other's substrates under long-term salinity stress we analyzed D1 phosphorylation in an *stn7* knockout mutant and the phosphorylation of Lhcb2 in an *stn8* mutant. Both mutants displayed an accumulation pattern of D1 and Lhcb2 similar to the wt plants in response to salt stress (Fig. S9 A). As expected, D1 was not phosphorylated in the *stn8* mutant and Lhcb2 – in *stn7*. Notably, in the *stn7* mutant we observed a higher level of D1 phosphorylation than in wt plants indicating that a disturbance in phosphorylation of the LHCII proteins also affects the phosphorylation and probably the turnover of D1 as well in the PSII core. The effect was not reciprocal as the phosphorylation of Lhcb2 was not affected in the stn8 mutant (Fig. S9 A). The *STN7* gene expression and STN7 protein level were stable during salinity in wt plants and the *snrk2.10* mutants (Fig. S9 B and Fig. S11), suggesting that the increased D1 phosphorylation in

snrk2.10 mutants is not related to the amount of STN7 in these plants. Importantly, the expression of *STN8* was similar in all plant lines studied (Fig. S11).

Supplemental references

Ashraf M, Harris PJC (2013) Photosynthesis under stressful environments: An overview. Photosynthetica 51: 163-190

Foyer CH, Shigeoka S (2011) Understanding Oxidative Stress and Antioxidant Functions to Enhance Photosynthesis. Plant Physiol 155: 93-100

Horton P (2012) Optimization of light harvesting and photoprotection: molecular mechanisms and physiological consequences. Phil Trans R Soc B 367: 3455–3465

Lichtenthaler HK (1987) Chlorophylls and carotenoids: Pigments of photosynthetic biomembranes. Methods in Enzymology 148: 350-382

Skupien J, Wojtowicz J, Kowalewska L, Mazur R, Garstka M, Gieczewska K, Mostowska A (2017) Dark-chilling induces substantial structural changes and modifies galactolipid and carotenoid composition during chloroplast biogenesis in cucumber (Cucumis sativus L.) cotyledons. Plant Physiol Biochem 111: 107-118

Szalonek, M., Sierpien, B., Rymaszewski, W., Gieczewska, K., Garstka, M., Lichocka, M., et al. (2015). Potato annexin STANN1 promotes drought tolerance and mitigates light stress in transgenic Solanum tuberosum L. plants. PLoS One 10:e0132683. doi: 10.1371/journal.pone.0132683

Supplemental Figures

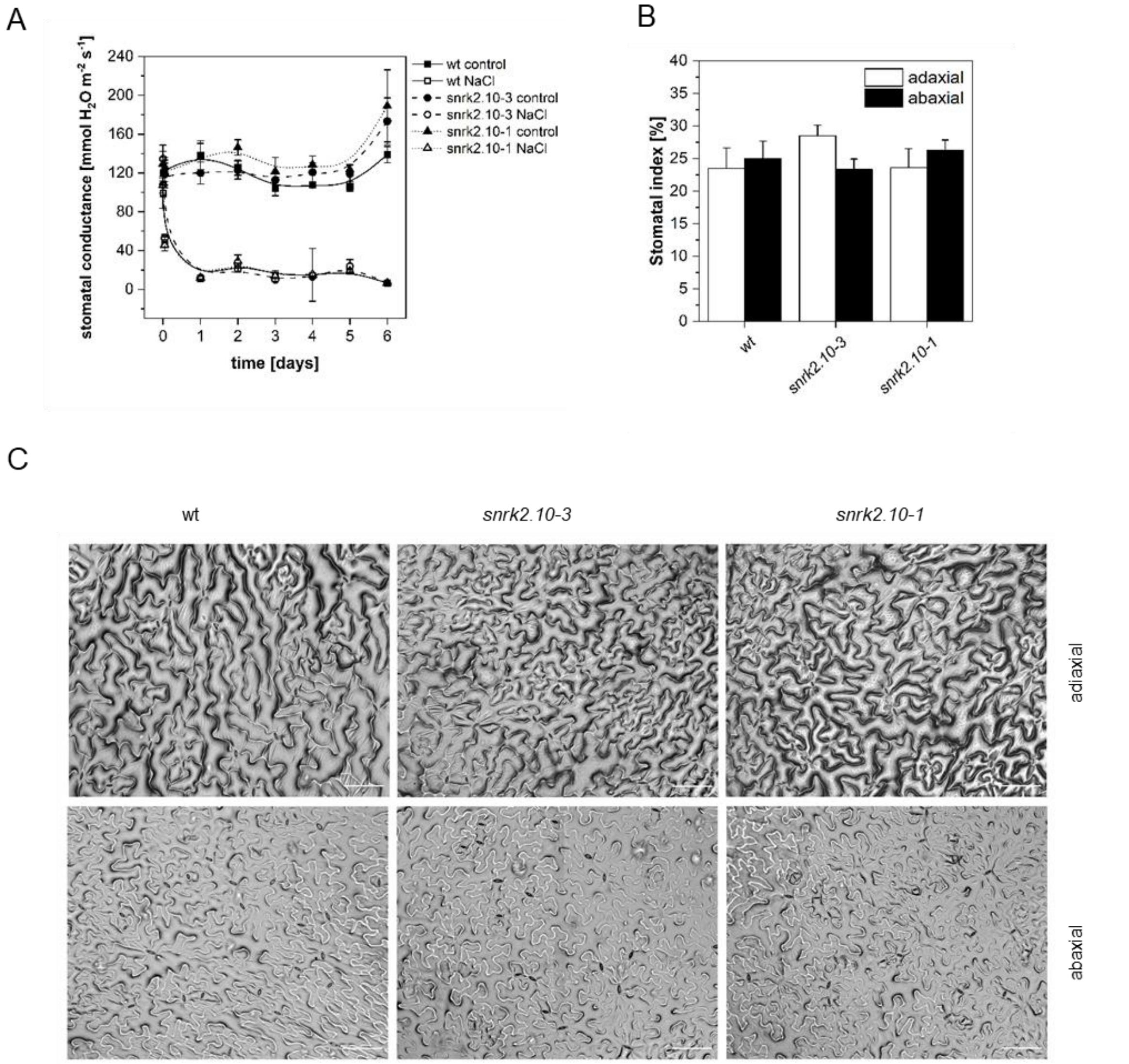

Supplemental Figure S1. Stomatal conductance and stomata index in wt and *snrk2.10* mutant lines.

(A) Stomatal conductance of fully developed leaves was measured by an AP4 automatic porometer. Graph presents one of four biological repetitions showing similar results. Fifeteen leaves were measured for each line and treatment in each experiment. (B) Stomata index. For each plant line stomata were counted for forty optical sections from adaxial and abaxial sides of the leaf. For (A) and (B) bars mean ± SD. Statistical significance of difference was determined using ANOVA followed by post hoc Tukeys test ($p<0.05$). (C) Representative DIC microphotographs of the density and distribution of the stomata on leaf surfaces. Pictures show adaxial (in the first row) and abaxial (in the second row) surface of Arabidopsis leaf epidermis: wild type Col-0 (a, d) and *snrk2.10-3* (b, e) and *snrk2.10-1* (c, f). Scale bars: 100 μm.

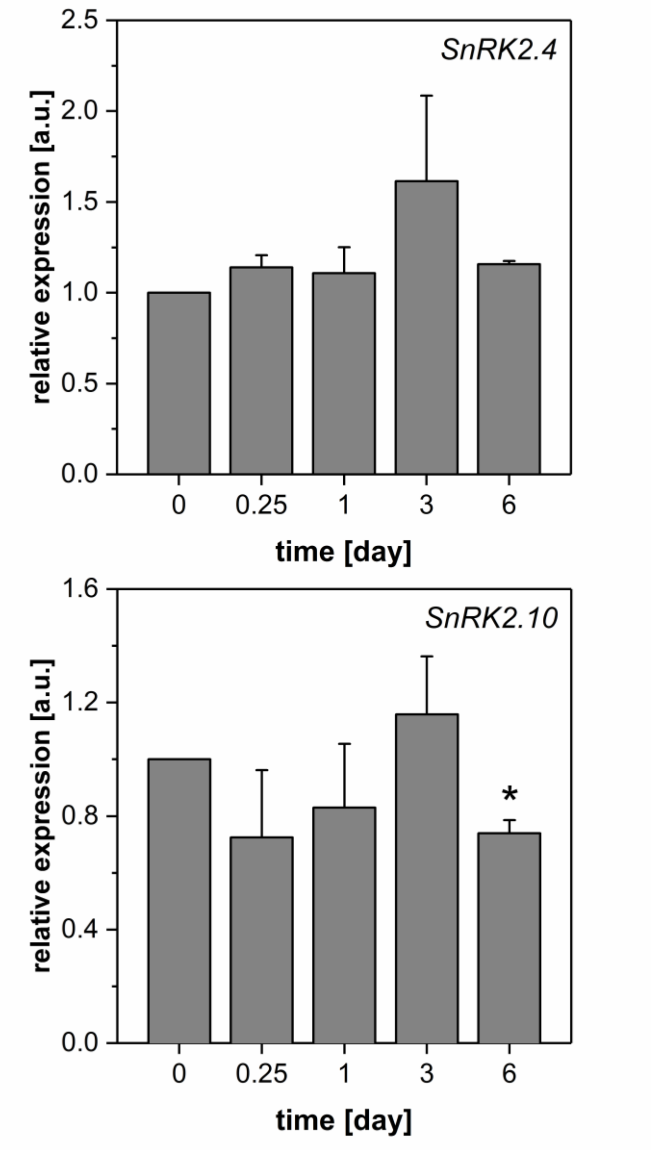

Supplemental Figure S2*. SnRK2.4 and SnRK2.10* expression under salt stress.

Transcript level was analyzed by RT-qPCR method in rosettes of wt plants treated with 150 mM NaCl for up to six days. Graphs show mean values ± SD from three independent biological repetitions with six plants used per treatment per line in each experiment. Statistical significance of difference determined using Student's t-test ($p<0.05$).

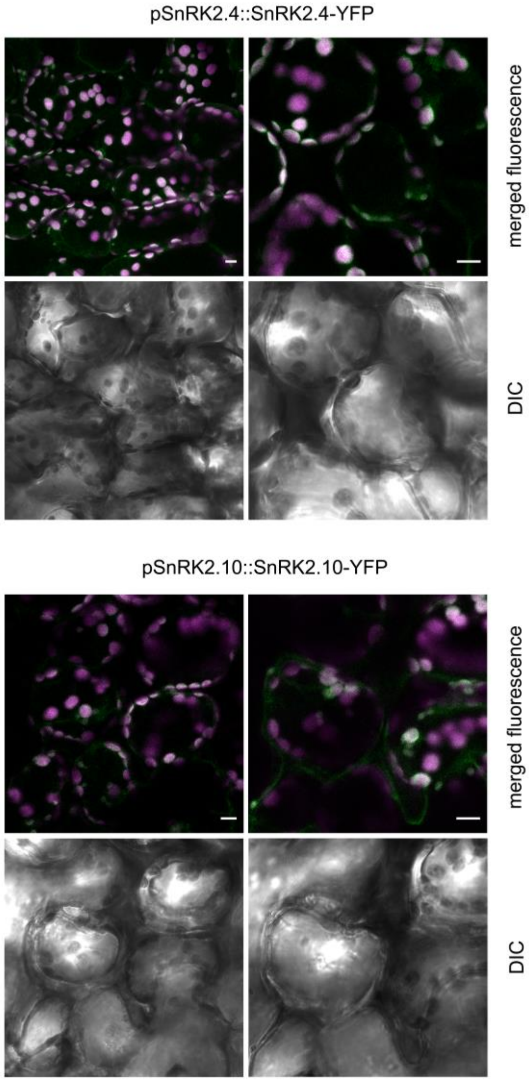

Supplemental Figure S3. **Localization of SnRK2.4 and SnRK2.10 in the leaf tissue.**

Localization of kinases in leaves was observed in transgenic plants expressing either pSnRK2.4::SnRK2.4-YFP or pSnRK2.10::SnRK2.10-YFP (McLouglin et al., 2012) using the fluorescent microscope. Representative images are z-series projections or single optical sections and show the cytosolic fluorescence signal of YFP (rendered in false green) and autofluorescence of chloroplasts (rendered in false magenta) in the palisade mesophyll cells. Brightfield reference images were collected in Nomarski contrast (DIC, differential interference contrast). Scale bars: 10 μm.

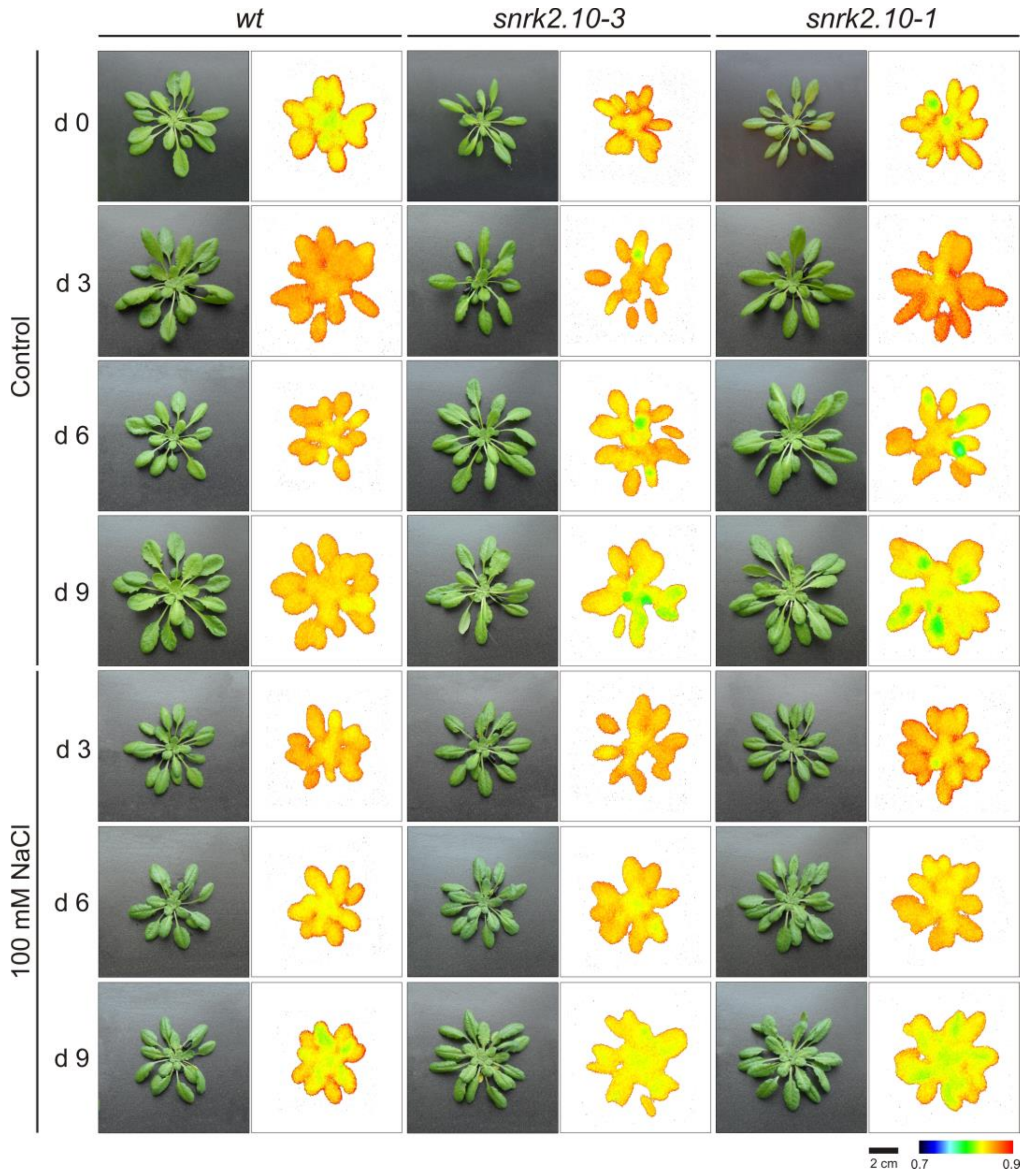

Supplemental Figure S4. Effect of 100 mM NaCl on chlorophyll *a* fluorescence distribution of wt and *snrk2.10* mutant plants.

Pictures present leaf morphology (left-hand columns) and F_V/F_M ratio (right-hand columns) for plants grown in standard medium (control) and plants treated with 100 mM NaCl for up to six days. The images are representative for at least four rosettes from each treatment.

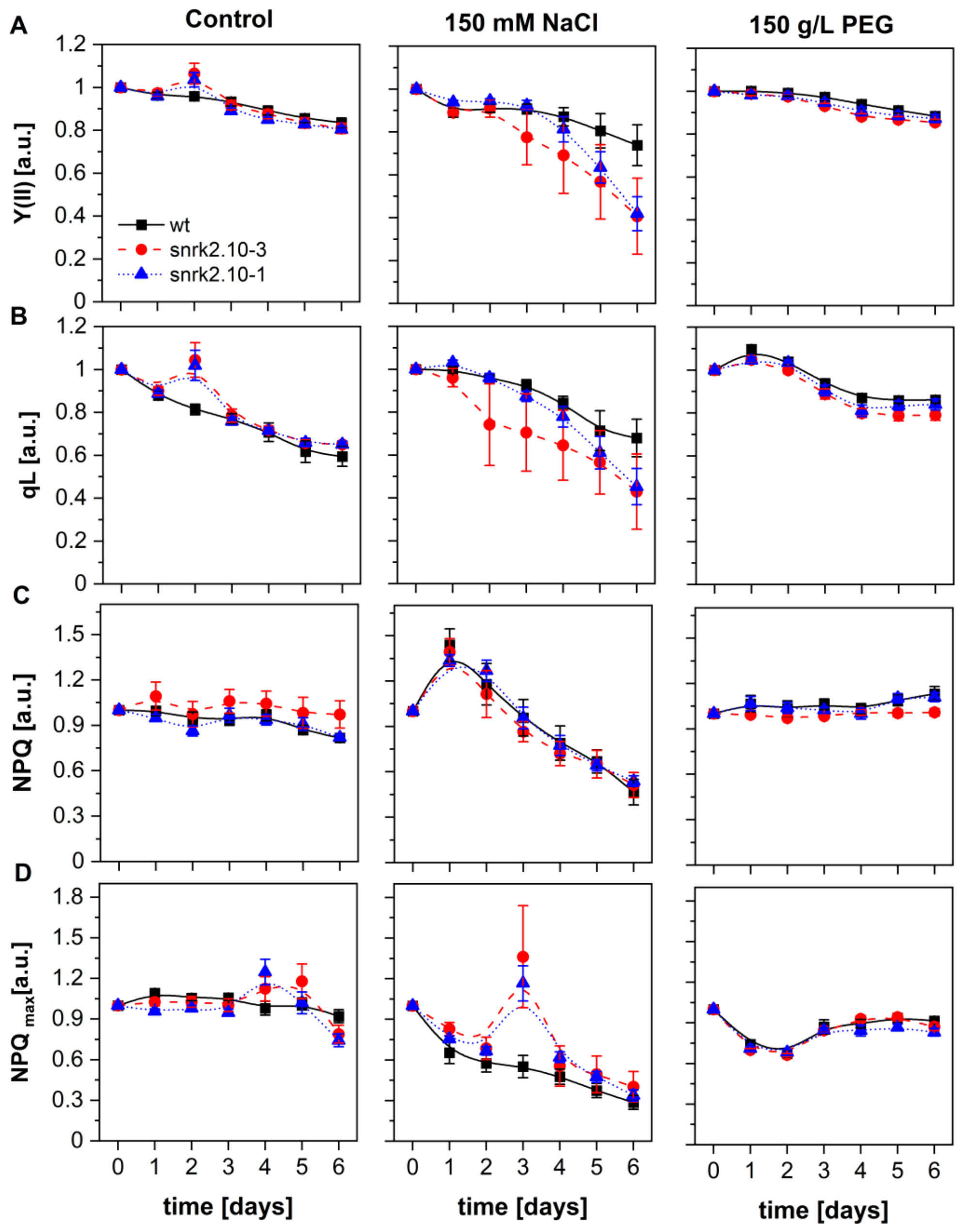

Supplemental Figure S5. Effect of 150 mM NaCl and 150 g/L PEG 8000 on efficiency of photosynthetic light reactions of wt and *snrk2.10* mutant plants under steady light intensity.

Photosynthetic parameters: Y(II) (A), qL (B), NPQ (C), and NPQ_{max} (D) were shown for steady-state light conditions i.e. the last saturation pulse (A-C) or maximal reached value (D) for wt and *snrk2.10* plants grown under control conditions and treated with 150 mM NaCl or 150 g/L PEG 8000. Data show mean values ± SE and are representative results for one from three independent experiments.

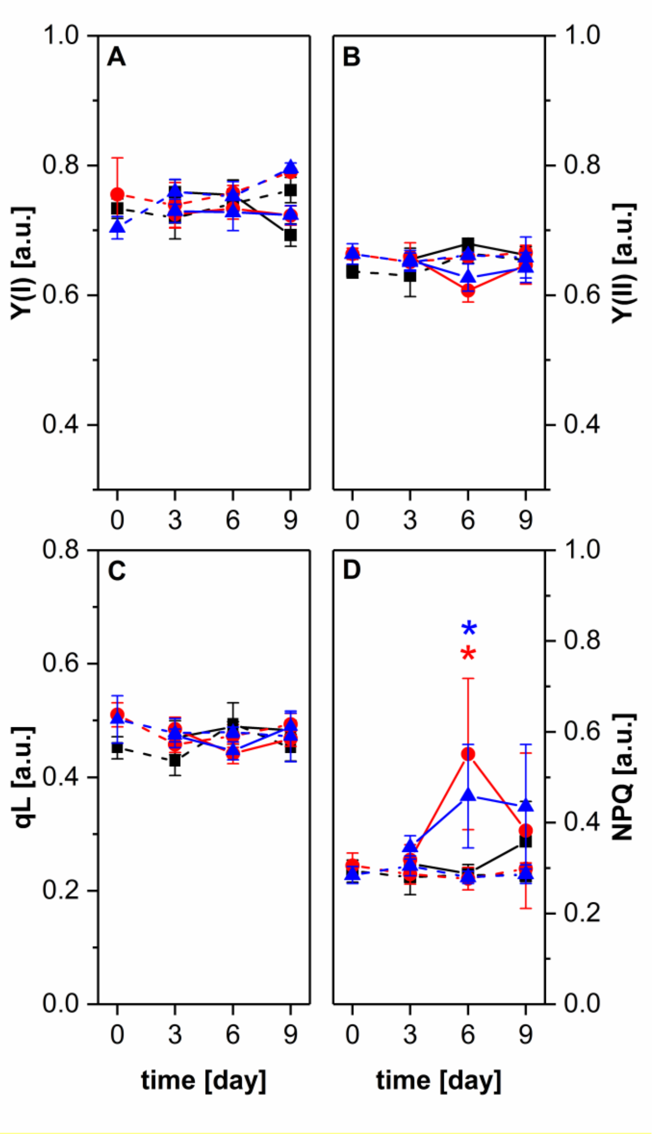

Supplemental Figure S6. Effect of 100 mM NaCl on efficiency of photosynthetic light reactions of wt and *snrk2.10* mutant plants under steady light intensity.

Photosynthetic parameters: Y(I) (A), Y(II) (B), qL (C) and NPQ (D) were determined under steady light conditions for wt (black squares), snrk2.10-3 (red circles) and snrk2.10-1 (blue triangles) plants grown under control conditions (dashed lines) and treated with 100 mM NaCl (solid lines). Data are means ± SD from three independent experiments. Statistical significance of difference was determined using ANOVA followed by post hoc Tukeys test ($p<0.05$). Asterisks mark statistical significance of difference between a mutant line and wt plants.

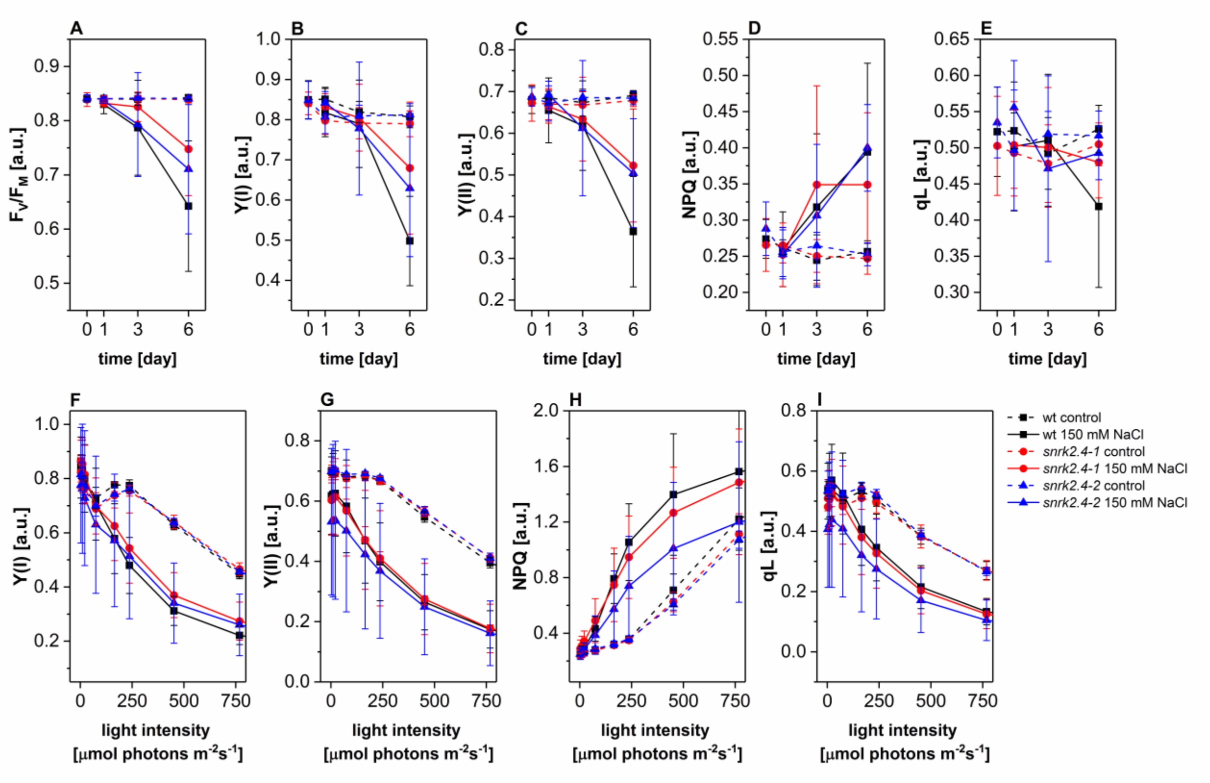

Supplemental Figure S7. Effect of salt stress on efficiency of photosynthetic light reactions of wt and *snrk2.4* mutant plants.

Photosynthetic parameters: Fv/Fm (A); Y(I) (B), Y(II) (C), NPQ (D) and qL (E) were determined under steady light conditions for wt (black squares), *snrk2.4-1* (red circles) and *snrk2.4-3* (blue triangles) plants grown under control conditions (dashed lines) and treated with 150 mM NaCl (solid lines). Photosynthetic parameters: Y(I) (F), Y(II) (G), NPQ (H) and qL (I) measured under increasing actinic light intensity for plants grown in control conditions (black dashed lines) and after treatment with 150 mM NaCl for three days (red solid lines). Data are means ± SD from three independent experiments.

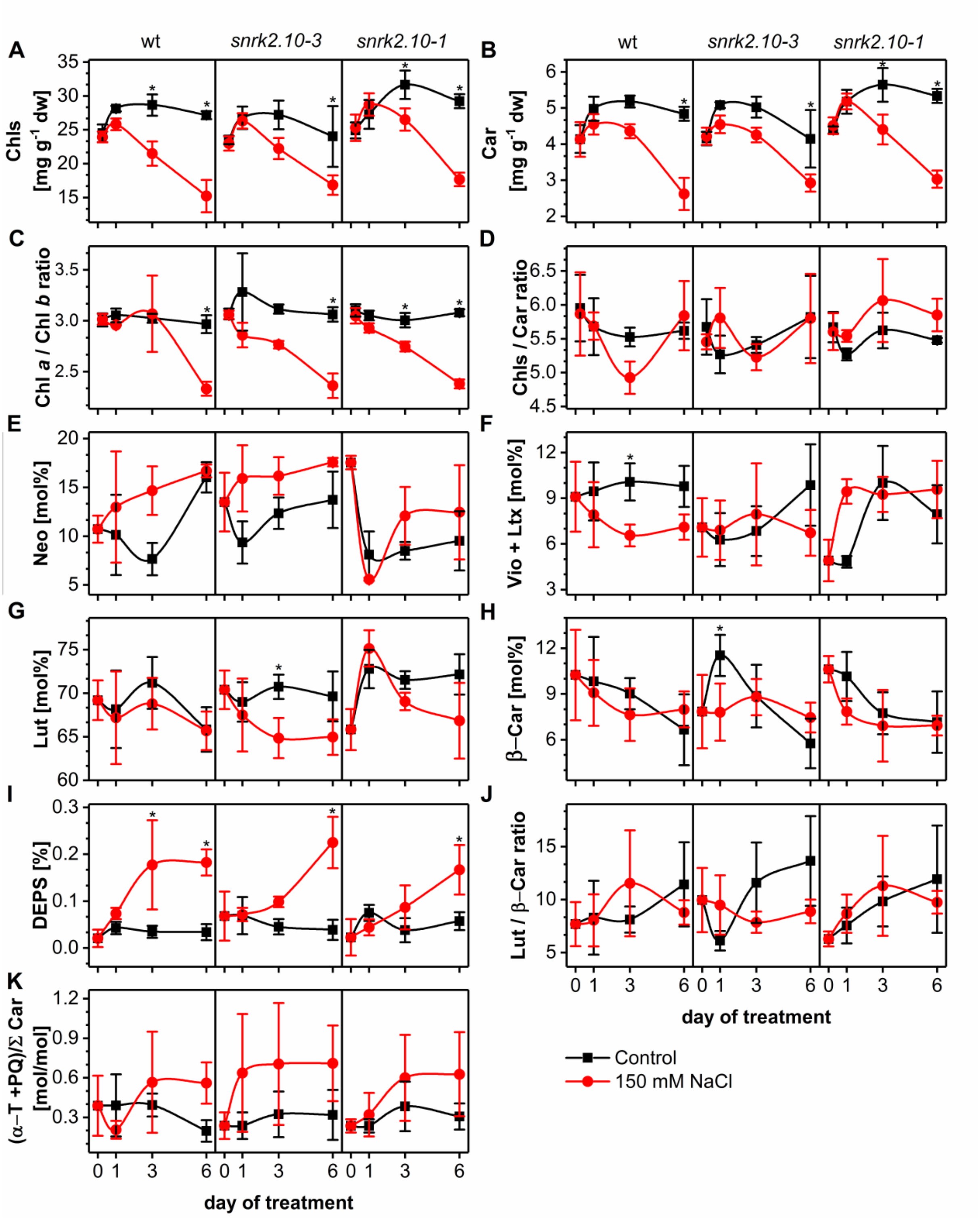

Supplemental Figure S8. Effect of salt stress on chlorophyll and carotenoid composition in wt and *snrk2.10* plants.

Rosettes from wt and *snrk2.10* mutant plants treated (red lines with circles) or not treated (black lines with squares) with 150 mM NaCl for up to six days were ground, pigments were extracted and their content determined as described in Materials and Methods. Content of

total chlorophylls (Chls) (A) and carotenoids (Car) (B), Chl a/Chl b (C) and chlorophyll/carotenoids ratio (D) were calculated basing on pigment absorption spectra and appropriate absorption coefficients. Content of neoxanthin (Neo) (E), a sum of violaxanthin and lutheoxanthin (Vio+Ltx) (F), Lutein (Lut) (G), β-carotene (β-Car) (H), the de-epoxidation status (DEPS) (I), Lut/ β-Car ratio (J) and 'antioxidant potential' (K) were calculated basing on UPLC chromatograms. The abundance of lutheoxanthin, which is converted non-enzymatically from violaxanthin did not exceed 2 mol % and therefore is presented as a sum with violaxanthin. DEPS was calculated as (Z + 0.5A)/(Z + A + V), where Z, A, and V are zeaxanthin, antheraxanthin, and violaxanthin, respectively. 'Antioxidant potential' was calculated as ratio of (α-tocopherol + plastoquinone) to the sum of all detected carotenoids. The data are mean ± SD for 4 to 9 independent experiments. Statistical significance of difference was determined using ANOVA followed by post hoc Tukeys test ($p<0.05$). Pairs of results marked with an asterisk differ significantly.

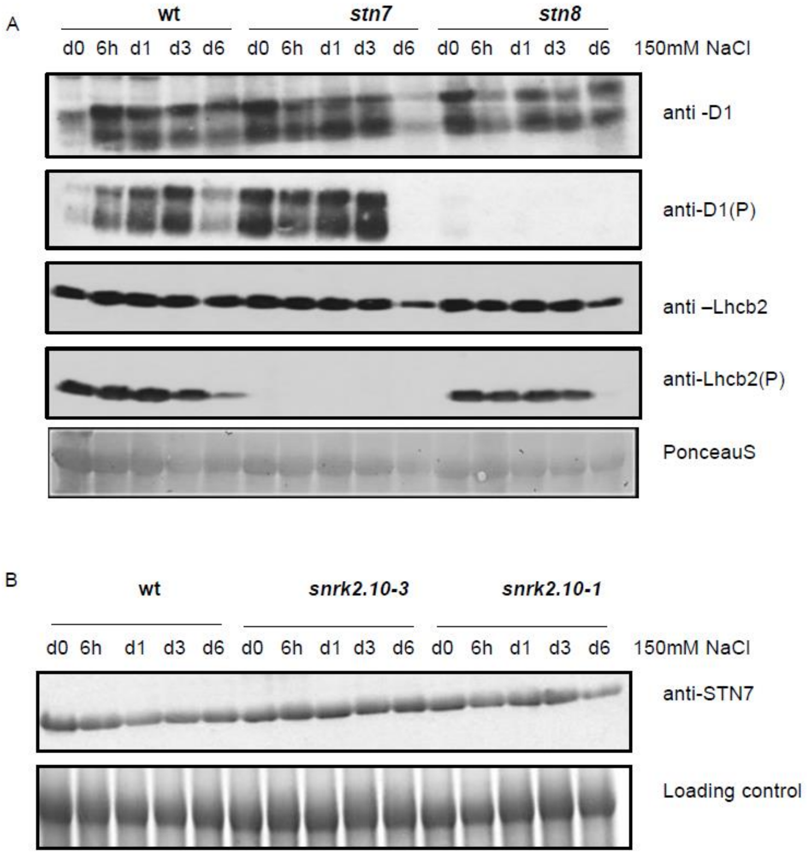

Supplemental Figure S9. STN7 and STN8 affect proteins levels and their phosphorylation status in plant chloroplasts under salt stress.

Proteins were extracted from rosettes of wild type, *stn7*, *stn8* and *snrk2.10* mutant plants treated with 150 mM NaCl for up to six days. Levels of D1 and Lhcb2 proteins and their phosphorylated forms (D1(P) and Lhcb2(P)) were determined by Western blotting with the use of specific antibodies (A). Level of STN7 protein was determined by Western blotting (B). Figure presents results one from three replicates of experiment showing similar results.

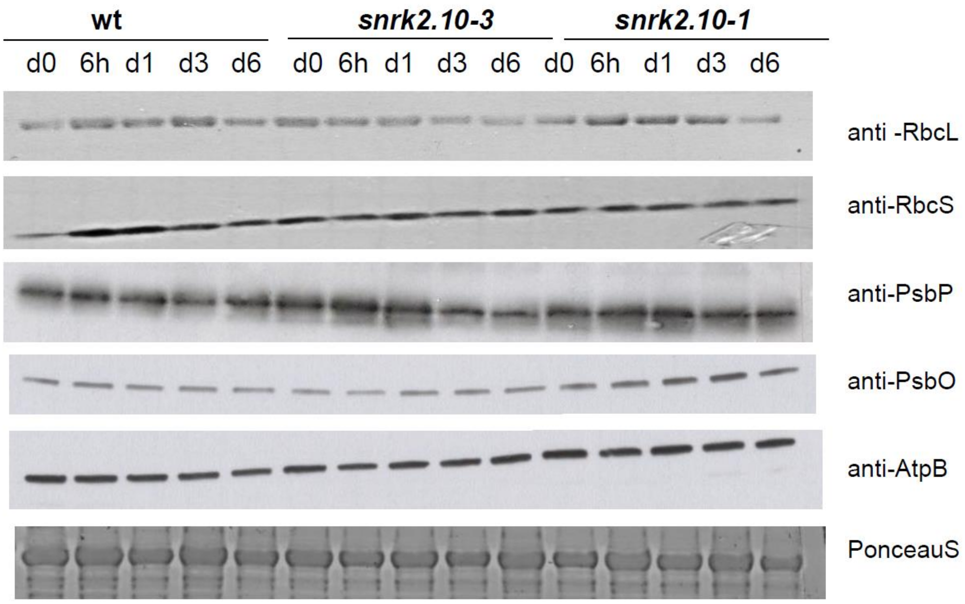

Supplemental Figure S10. Levels of photosynthesis-related proteins in wt, *snrk2.10-3* and *snrk2.10-1* plant lines under long term salinity.

Proteins were extracted from rosettes of wild type, *snrk2.10-3* and *snrk2.10-1* insertion mutants treated with 150 mM NaCl for up to six days. The RbcL, RbcS, PsbP, PsbO and AtpB levels were determined by Western blotting with specific antibodies. Figure presents results of one of three replicates of experiment showing similar results.

AGI	Gene name	wt					*snrk2.10-3*					*snrk2.10-1*				
		d0	6h	d1	d3	d6	d0	6h	d1	d3	d6	d0	6h	d1	d3	d6
ATCG00490	*RBCL*	1.00 ± 0	0.83 ± 0.07	0.72 ± 0.03	0.47 ± 0.06	0.23 ± 0	0.82 ± 0.26	0.65 ± 0	0.85 ± 0.06	0.41 ± 0.05	0.22 ± 0	0.88 ± 0.17	0.90 ± 0.14	0.75 ± 0.05	0.39 ± 0.01	0.22 ± 0.06
AT1G67090	*RBCS1A*	1.00 ± 0	0.53 ± 0.06	0.27 ± 0.08	0.44 ± 0.13	0.35 ± 0.18	0.91 ± 0.2	0.44 ± 0.15	0.32 ± 0.13	0.48 ± 0.16	0.33 ± 0.19	0.91 ± 0.06	0.58 ± 0.25	0.24 ± 0.07	0.48 ± 0.22	0.40 ± 0.23
ATCG00020	*PSBA*	1.00 ± 0	0.89 ± 0.21	0.60 ± 0.07	0.60 ± 0.19	0.44 ± 0.09	0.55 ± 0	0.59 ± 0.10	0.88 ± 0.06	0.56 ± 0.18	0.61 ± 0.21	0.64 ± 0.15	0.65 ± 0.21	0.68 ± 0.18	0.23 ± 0.09	0.44 ± 0.10
AT4G04640	*ATPC1*	1.00 ± 0	1.15 ± 0.15	0.39 ± 0.07	0.47 ± 0.03	0.26 ± 0.09	1.17 ± 0.13	0.82 ± 0.32	0.51 ± 0.29	0.47 ± 0.15	0.22 ± 0.07	1.14 ± 0.21	1.04 ± 0.14	0.39 ± 0.11	0.48 ± 0.04	0.29 ± 0.09
AT1G06680	*PSBP-1*	1.00 ± 0	0.78 ± 0.22	0.38 ± 0.14	0.42 ± 0.24	0.27 ± 0.18	0.84 ± 0.12	0.64 ± 0.24	0.46 ± 0.26	0.41 ± 0.19	0.16 ± 0.1	1.04 ± 0.06	0.73 ± 0.18	0.32 ± 0.14	0.29 ± 0.07	0.25 ± 0.16
AT4G21280	*PSBQ-1*	1.00 ± 0	0.59 ± 0.01	0.23 ± 0.07	0.18 ± 0.01	0.11 ± 0.01	0.89 ± 0.01	0.41 ± 0.04	0.15 ± 0.07	0.19 ± 0.05	0.09 ± 0.04	0.86 ± 0.10	0.53 ± 0.01	0.22 ± 0.09	0.22 ± 0.05	0.11 ± 0.01
AT3G27690	*LHCB2.4*	1.00 ± 0	0.34 ± 0.25	0.18 ± 0.05	0.12 ± 0.07	0.13 ± 0.15	0.92 ± 0.08	0.29 ± 0.1	0.15 ± 0.06	0.13 ± 0.06	0.03 ± 0.03	0.98 ± 0.06	0.35 ± 0.19	0.18 ± 0.14	0.09 ± 0.03	0.06 ± 0.07
AT1G12900	*GAPA-2*	1.00 ± 0	0.35 ± 0.15	0.12 ± 0	0.24 ± 0.05	0.12 ± 0.02	0.80 ± 0.39	0.34 ± 0.09	0.25 ± 0.09	0.33 ± 0.01	0.10 ± 0.01	0.88 ± 0.28	0.35 ± 0.09	0.18 ± 0.01	0.21 ± 0.10	0.13 ± 0.01
AT2G20570	*GLK1*	1.00 ± 0	0.71 ± 0.20	0.71 ± 0.22	0.72 ± 0.3	0.41 ± 0.30	1.05 ± 0.13	0.72 ± 0.14	0.62 ± 0.08	0.60 ± 0.04	0.32 ± 0.23	1.19 ± 0.18	0.86 ± 0.16	0.81 ± 0.14	0.68 ± 0.08	0.42 ± 0.37
AT5G44190	*GLK2*	1.00 ± 0	0.47 ± 0.11	0.29 ± 0.06	0.51 ± 0.03	0.46 ± 0.17	0.90 ± 0.11	0.43 ± 0.08	0.26 ± 0.02	0.51 ± 0	0.40 ± 0.01	1.08 ± 0.27	0.46 ± 0.1	0.34 ± 0.04	0.58 ± 0.04	0.51 ± 0.13
AT1G68830	*STN7*	1.00 ± 0	0.90 ± 0.07	1.03 ± 0.03	1.1 ± 0.05	1.41 ± 0.01	1.33 ± 0.09	0.99 ± 0.03	1.34 ± 0.13	1.48 ± 0.04	1.20 ± 0.08	1.45 ± 0.00	1.21 ± 0.03	1.15 ± 0.00	1.47 ± 0.06	1.42 ± 0.18
AT5G01920	*STN8*	1.00 ± 0	0.56 ± 0.01	0.60 ± 0.01	0.93 ± 0.03	1.00 ± 0.02	0.85 ± 0.03	0.72 ± 0.01	0.54 ± 0.02	0.99 ± 0.04	1.21 ± 0.06	1.99 ± 0.00	0.78 ± 0.07	0.81 ± 0.02	1.06 ± 0.04	1.07 ± 0.07

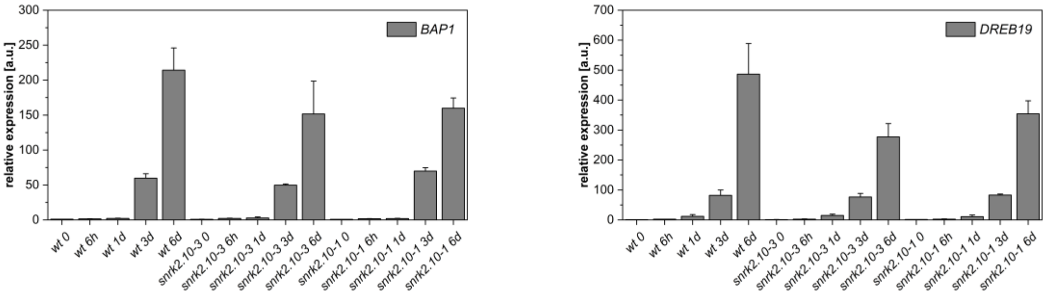

Supplemental Figure S11. Expression of photosynthesis- and stress-related genes under salinity.

Transcript level was monitored by RT-qPCR in rosettes of five-week-old plants, not treated or treated with 150 mM NaCl for 0-6 days. Transcript level of a gene is plotted relative to that in non-treated wild-type plants (Col-0). Shown are representative results from independent biological replicates, each with 8-10 plants from each line per treatment.

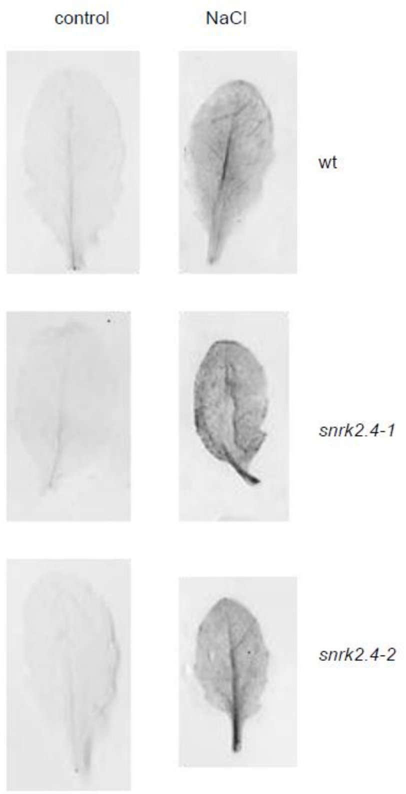

Supplemental Figure S12. Accumulation of H_2O_2 in *A. thaliana* leaves of *snrk2.4* mutant plants under long-term salinity.

Six-week-old plants of wild type and *snrk2.4* mutant were treated with 150 mM NaCl or kept in control media for six days. Leaves of the similar age were collected at indicated times and stained for H_2O_2 with DAB. After removal of photosynthetic and nonphotosynthetic pigments leaves were photographed to show DAB staining. Figure presents results one from three replicates of experiment showing similar results with eight to ten leaves used per line and treatment in every biological repetition.

Supplemental Table S1. List of primers used in this study.

AGI	Gene name	Primers
AT1G10940	SnRK2.4	F - CTCGACAACCCTAATTCCAAAC
		R - GCTGCTAGAACTGGACTTGA
At1G60940	SnRK2.10	F -TGGAGTGAGAATGTCGGGTTCT
		R - TCCCGTATATTTGTCACCAACTCG
ATCG00490	*RBCL*	F - TCGGTGGAGGAACTTTAGGC
		R - TGCAAGATCACGTCCCTCAT
AT1G67090	*RBCS1A*	F - AATTTCCGGACTTAACGTTTGTTT
		R - CATCAGACAGTTGAGAATCCGATAGA
ATCG00020	*PSBA*	F - GTGCCATTATTCCTACTTCTG
		R - AGAGATTCCTAGAGGCATACC
AT4G04640	*ATPC1*	F - TCGTGGATTATGTGGTGGATTC
		R - CCTTCTTGCCCACGCTAAT
AT1G06680	*PSBP-1*	F - CTCAGGAAGTTGGTGGGAAA
		R - ATTCACGGTTGCTGTGATCA
AT4G21280	*PSBQ-1*	F - ATCAAAGTTGGCCCTCCTCC
		R - TCAATGCCAATGCAAAGTCTC
AT3G27690	*LHCB2.4*	F - GCCATCCAACGATCTCCTC
		R - TGGTCCGTACCAGATGCTC
AT1G12900	*GAPA-2*	F - GCTTAGTCGTGCAAGTCTCCAAGAA
		R - GCTCATCACAGACGTCAAGGATACC
AT2G20570	*GLK1*	F - CATCCAATGCATAACGGGACGACT
		R - TGGCGGTGCTCTAAATCTCGTAGC
AT5G44190	*GLK2*	F - TCCCATCGACATTCATCCCTCGAA
		R - GGCGGTTTCAGTCCCAAAGGAA
AT1G68830	*STN7*	F - TAGTGGAGCCTCGTGCAAGC
		R - ACGGACAACGCGAGCAATCC
AT5G01920	*STN8*	F - CTTAATAGGTGGCGGGAAG
		R - AGAGAACCTCTCTCGGATATG
AT1G13320	*PDF2*	F - TAACGTGGCCAAAATGATGC
		R - GTTCTCCACAACCGCTTGGT
AT5G25760	*PEX4*	F - CTGCGACTCAGGGAATCTTCTAA
		R - TTGTGCCATTGAATTGAACCC
